# Physiological and biochemical responses of twelve autochthonous grapevine varieties to summer stress in the Douro Demarcated Region

**DOI:** 10.3389/fpls.2026.1757496

**Published:** 2026-02-05

**Authors:** Maria Gaspar, Márcia Carvalho, Miguel Baltazar, Helena Ferreira, Joana Valente, Fernando Alves, Isaura Castro, Berta Gonçalves

**Affiliations:** 1 Centre for the Research and Technology of Agro-Environmental and Biological Sciences (CITAB), University of Trás-os-Montes e Alto Douro (UTAD), Vila Real, Portugal; 2 Institute for Innovation, Capacity Building and Sustainability of Agri-food Production (Inov4Agro), University of Trás-os-Montes e Alto Douro (UTAD), Vila Real, Portugal; 3 Symington Family Estates, Vinhos SA, Vila Nova de Gaia, Portugal

**Keywords:** antioxidant activity, autochthonous varieties, grapevine varietal diversity, leaf gas exchange, summer stress

## Abstract

Grapevine (*Vitis vinifera* L.) is one of the most cultivated crops in Portugal and worldwide and plays an important role in the Mediterranean countries’ economies. In the last decade, this region has faced increasing summer stress that negatively impacts plant growth and development and reduces crop’s yield and quality. These challenges can be addressed by exploring grapevine varietal diversity to identify promising genotypes capable of coping with summer stress. In this study, twelve different autochthonous varieties grown in the *Douro Superior* sub-region from the Douro Demarcated Region were studied in two different phenological stages (veraison and maturity) and two consecutive growing seasons (2023 and 2024). Measurements included leaf gas exchange parameters (in the morning and midday), lipid peroxidation analysis, determination of the contents of photosynthetic pigments, total soluble sugars, starch, bioactive compounds - including total phenolics, flavonoids, and ortho-diphenols -, and of the antioxidant activity using ABTS, DPPH, and FRAP assays. The data obtained revealed that ‘Tinta Roriz’ and ‘Alicante Bouschet’ presented photosynthetic activity, water use efficiency and pigment levels, suggestive of tolerance to summer stress. On the other hand, ‘Mourisco de Semente’, ‘Malvasia Preta’, and ‘Touriga Fêmea’ revealed lower antioxidant activity, and photosynthetic capacity, indicative of higher vulnerability to stress conditions. The remaining varieties presented an intermediate summer stress tolerance or a growing season-tolerance variability. These findings highlight the diversity of responses of different grapevine varieties to summer stress and reinforce the importance of identifying stress-resilient genotypes.

## 1 Introduction

*Vitis vinifera* L. is one of the most important fruit crops worldwide (Baltazar et al., 2025a) and among the most widespread cultivated species due to its economic importance (Carvalho et al., 2024). Viticulture is an ancient agricultural activity with wine and table grapes being the most distinctive products (Prada et al., 2024). The development and growth of grapevines, as well as their physiological processes, plant quality, and yield, are directly influenced by the interaction of climate, soil, geography, grapevine variety, and cultural practices (Fraga et al., 2014; Monteiro et al., 2024c; Prada et al., 2024; Baltazar et al., 2025a). Vineyards worldwide have experienced several climatic disruptions due to climate change (OIV, 2025). Mediterranean countries, namely Portugal and Greece, report five-year average wine production losses up to 30% (Baltazar et al., 2025a; OIV, 2025). These regions have a Mediterranean climate that is characterized by two distinct seasons: cool, wet winters and hot, dry summers (Prada et al., 2024; OIV, 2025). The Mediterranean region is classified as a significant climate change hotspot due to reporting an increase in temperature and frequency of extreme weather events, prolonged periods of severe drought, and higher levels of ultraviolet radiation. These changes will have significant effects on agriculture, as well as food security and safety (Schultz, 2000; Dinis et al., 2022; Prada et al., 2024; Baltazar et al., 2025a). Portuguese vineyards, including the Douro Demarcated Region (DDR) in the northeast of Portugal, have also been facing the effects of climate change, with increasingly drier and warmer conditions, and rising frequency and intensity of extreme weather events (Monteiro et al., 2024c). The DDR has a unique terroir and Mediterranean climate conditions (Monteiro et al., 2023; Monteiro et al., 2024c; Monteiro et al., 2024a; Monteiro et al., 2024b). The ‘Douro Superior’ subregion has a specific microclimate that has been experiencing an increasing combination of stress factors, such as higher temperatures and radiation, and more severe water deficits (Carvalho et al., 2024; Monteiro et al., 2024a). This type of climate change has been classified as summer stress and affects grapevines differently depending on the region. However, a high level of summer stress can hinder grapevine development and decrease grape yield and quality, generating biological responses that may threaten its survivability (Biasi et al., 2019; Baltazar et al., 2025a). When grapevines are subjected to summer stress, photosynthesis is one of the first biological processes to be affected, decreasing due to the extreme sensitivity of grapevine leaves to heat and radiation stress. To prevent water loss, leaves tend to close the stomata, leading to changes in gas exchange rates. Therefore, analyzing this parameter by measuring leaf gas exchanges is essential when selecting for varietal adaptability (Brito et al., 2024; Baltazar et al., 2025a). Other leaf components are also affected by the plant’s exposure to summer stress as part of its response to mitigate negative effects. Total soluble sugar and starch in grapevine leaves commonly suffer changes under stress, as these compounds are synthesized during photosynthesis and play key roles in energy storage, organic compound production, and the formation of cellulose and hemicellulose. However, high temperatures, particularly at night, prevent their transport to the berries, leading to an accumulation of these compounds in the leaves (Zufferey et al., 2012; Tombesi et al., 2019). Some photosynthetic pigments, such as carotenoids, tend to increase in leaves when the plant is under summer stress, as they play a protective role by mitigating oxidative stress and acting as quenchers of chlorophyll molecules. This response is known to be variety-dependent (Carvalho et al., 2016). Similarly, phenolic compounds, such as total phenolics, flavonoids, and *ortho*-diphenols, vary among grapevine varieties and are crucial for grape quality. Another important function of these compounds is the plant’s defense mechanisms against summer stress (Luzio et al., 2021). The exposure of plants to summer stress negatively impacts their metabolism by causing oxidative damage to proteins, lipids, and nucleic acids. The presence of this oxidative stress, which can be analyzed using thiobarbituric acid reactive substances (TBARS), induces the activation of antioxidant enzymes (Tzortzakis et al., 2020). This activation promotes changes in antioxidant activity when the plant is exposed to summer stress, making it a very important parameter to analyze (Monteiro et al., 2024).

The increase in summer stress and climate change, in general, highlights the urgent need for research on grapevine adaptation strategies. These strategies include studying the intraspecific diversity of grapevines, as has been reported different grapevine varieties have different responses to abiotic and biotic stress conditions (Biasi et al., 2019; Morales-Castilla et al., 2020; Serrano et al., 2022; Carvalho et al., 2024). Despite these varieties belonging to the same species, they present significant genomic and phenotypic plasticity. Their study can help to identify varieties better able to face climate changes, while also to select desirable traits in wine production. The use of optimally adapted grapevine varieties for each growing region is a long-term adaptation strategy and may be one of the most effective measures for enhancing the sustainability of the viticulture sector (Morales-Castilla et al., 2020; Carvalho et al., 2024; Baltazar et al., 2025a). The relationship between net photosynthesis and stomatal conductance, and carbon isotope discrimination in grapes, have been considered reliable physiological indicators for selecting efficient genotypes to future environmental conditions within cv. ‘Grenache’ (Buesa et al., 2021). ‘Bobal’, ‘Garnacha Peluda’, ‘Garnacha Tinta’, ‘Mazuela’, and ‘Moribel’ cultivars have been considered well responders to drought conditions, maintaining high yields and must quality through assessment of carbon isotope (Biasi et al., 2019; Morales-Castilla et al., 2020; Serrano et al., 2022; Carvalho et al., 2024). Color attributes, phenolic composition, and antioxidant potential of 24 white grape varieties over two consecutive growing seasons in the Douro Demarcated Region revealed notable varietal and year-to-year differences in both phenolic content and antioxidant potential with the cvs. as ‘Cerceal Branco’ and ‘Moscatel Galego Branco’ showing significant increases in total phenolic content and antioxidant activity under warmer and drier conditions, suggesting a robust physiological response to abiotic stress (Baltazar et al., 2025b). Baltazar et al noticed increased anthocyanin content in a warmer and drier season in cvs. such as ‘Tinto Cão’ and ‘Touriga Franca’ and decline in the cv. ‘Vinhão’, highlighting differential varietal responses to abiotic stress.

This study aimed to evaluate the behavior of 12 different grapevine varieties grown in a Mediterranean climate based on physiological and biochemical approaches during two consecutive growing seasons (2023 and 2024). The analysis included leaf gas exchange measurements, lipid peroxidation analysis, photosynthetic pigments, total soluble sugar and starch, bioactive compounds such as total phenolics, flavonoids, and *ortho*-diphenols contents, and the assessment of the antioxidant activity using ABTS, DPPH, and FRAP assays.

## 2 Material and methods

### 2.1 Plant material and sampling

**Figure 1:**
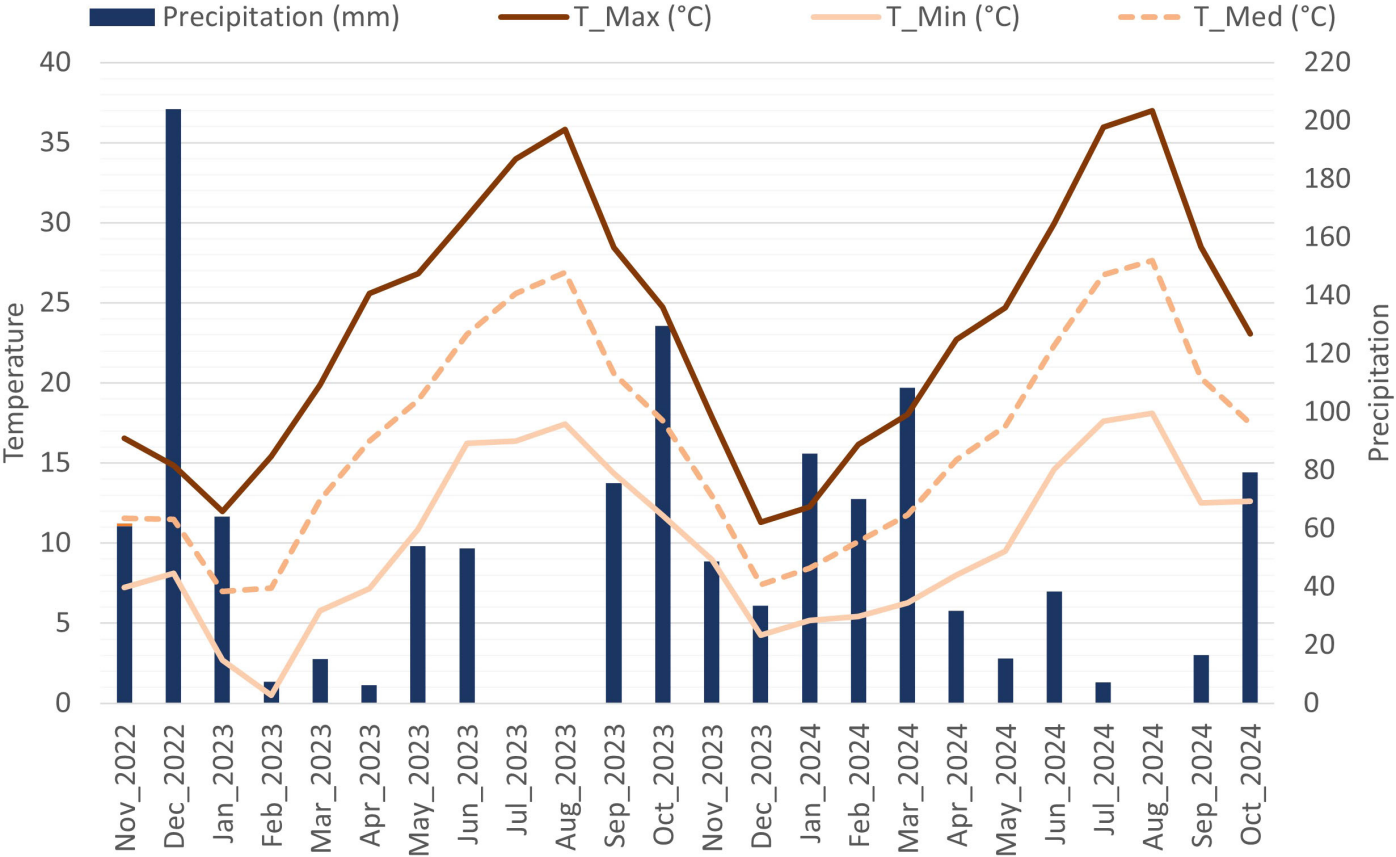
Monthly mean temperatures and precipitation ocurred during the 2023 and 2024 growing seasons in the vineyard Quinta do Ataíde in the Douro Superior sub-region. Precipitation (mm); Maximum temperature – T_Max (°C); Minimum temperature - T_Min (°C) and Mean temperature – T_Med (°C).

The experimental trial was conducted at Quinta do Ataíde, a commercial vineyard planted in 2013, located in the *Douro Superior* (Upper Douro) sub-region of the Douro Demarcated Region, Vila Flor, Portugal (41°15’03.3’’N 7°06’38.7’’W, 160 m above sea level). Twelve different autochthonous varieties (‘Touriga Nacional’, ‘Touriga Franca’, ‘Tinta Roriz’, ‘Tinto Cão’, ‘Tinta Barroca’, ‘Trincadeira’, ‘Vinhão’, ‘Donzelinho Tinto’, ‘Alicante Bouschet’, ‘Touriga Fêmea’, ‘Malvasia Preta’, and ‘Mourisco de Semente’) of *Vitis vinifera* cv. grafted on 196–17 C rootstock were studied throughout two growing seasons (2023 and 2024) and two phenological stages (veraison and maturity). The vineyard was arranged in four aligned blocks each composed of parallel rows of 25 vines per cultivar. The row spacing was 2.10 m and vine spacing was 0.9 m and the vines were trained to unilateral Royat Cordon, with vertical shoot positioning (VSP) in an east–southeast to west–northwest orientation. The vines were grown following the standard cultural practices usually employed by local wine producers. The collection was drip-irrigated, with 25% replacement of the ETc (crop evapotranspiration), that corresponds to a regulated deficit irrigation (RDI) approach, from bunch closure until one week before harvest. Crop evapotranspiration (ETc) for grapevine was calculated following the standard FAO methodology, as the product of reference evapotranspiration (ET_0_) and the grapevine crop coefficient (Kc), i.e., ETc = ET_0_ × Kc. Reference evapotranspiration was estimated using the FAO Penman–Monteith equation, and Kc value was selected according to grapevine phenological stage, as widely reported in the literature. In 2023, the irrigation period extended from 18 July to 29 August, with a total irrigation amount of 53.4 mm per block. In 2024, irrigation was applied from 23 July to 3 September, totaling 55.2 per block. This parcel is characterized by cold winters, with several days of temperatures below 0°C, and dry hot summers. The monthly temperature values and precipitation were calculated via a weather station located near the experimental site (daily recorded) and are shown in **Figure 1**.

During the first growing season (November 2022–October 2023), the mean daily temperature was 16.6°C reaching a peak monthly average of 35.8°C in August. Precipitation during this season was 670 mm. The second growing season (November 2023–October 2024) had a similar mean daily temperature of 16.5°C, but a higher peak monthly average of 37.0°C in August. Moreover, this season received less precipitation, totaling 536 mm.

For the biochemical parameters’ evaluation, one fully developed leaf from shoots middle section in five different grapevines plants was collected and pooled at veraison and maturity, during morning period. The procedure was repeated to the four replicates from each variety. These samples were immediately stored on dry ice for transport to the lab, where they were kept at - 80°C until grounded into fine powder using liquid nitrogen and then were sub-divided to the several biochemical parameters analysis.

### 2.2 Gas exchange measurements

Leaf gas exchange parameters were determined at veraison and maturity in the two years of the trial (2023 and 2024) with a portable infrared gas analyzer system (IRGA) (LCpro+, ADC, Hoddesdon, UK). Net CO_2_ assimilation rate (A), stomatal conductance (g_s_), intercellular CO_2_ concentration (C_i_), transpiration rate (E), and intrinsic water use efficiency (A/g_s_) were estimated according to the equations described by von Caemmerer and Farquhar (1981) (Von Caemmerer and Farquhar, 1981). These measurements were conducted at Quinta do Ataíde (under natural conditions) and were used adult leaves (n = 4 per variety), on sunny days, in the morning (8 am - 10 am), and at solar noon (1 pm - 2 pm). Veraison data were collected on July 26^th^ 2023 and 25^th^ July 2024, while maturity data were collected on 31^st^ August 2023 and 5^th^ September 2024.

### 2.3 Lipid peroxidation

To determine the oxidative damage of lipids in 10 mg of grapevine leaves (n=4 per year, per phenological stage) and mixed a solution of trichloroacetic acid (TCA) 20% to obtain the extract. The TBARS reagent (thiobarbituric acid reactive substances) protocol was used, following the microscale method described by Monteiro et al (Monteiro et al., 2024b). The measurements were performed in triplicate and was used the microplate reader. TBARS concentration was expressed as nmols TBARS per g of fresh weight (nmol g^-1^ FW).

### 2.4 Photosynthetic pigments determination

To determine the content of photosynthetic pigments, 10 mg of fresh leaves (FW) was used for each sample (n=4 per year, per phenological stage). The extraction was performed using acetone 80% (v/v). The quantification of chlorophyll *a* and *b*, total chlorophyll, and carotenoids was performed in triplicate according to the methods described by Monteiro et al. (2024b) using a microplate reader. These pigments were determined following the equations described by Mackinney (1941):


$$Chlorophylla=\left(12.7\times Abs_{663}-2.69\times Abs_{645}\right)/1000$$

$$Chlorophyllb=\left(22.9\times Abs_{645}-4.68\times Abs_{663}\right)/1000$$

$$Total\;chlorophyll=\left(0.0202\times Abs_{645}+0.00802\times Abs_{663}\right)$$

$$Carotenoids=\left(\left(1000\times Abs_{470}-1.82\times Cl_{a}-85.02\times Cl_{b}\right)/198\right)\times 0.001$$

The photosynthetic pigment results were expressed as mg per g of fresh weight (mg g^−1^ FW).

### 2.5 Total soluble sugars and starch determination

To determine the content of total leaf soluble sugars and leaf starch, the methodology described by Monteiro et al. (2024b) was followed. A total of 10 mg of fresh leaves (n=4 per year, per phenological stage) was used, and three technical replicates were performed. A glucose calibration curve (r^2^ soluble sugars = 0.9861; r^2^ starch = 0.9352) was used to express the results in mg glucose g^-1^ of fresh weight (mg g^-1^FW).

### 2.6 Determination of bioactive compounds and antioxidant activity

To prepare the sample extract (n=4 per year, per phenological stage), 40 mg of fresh leaves were weighed, and then 1.5 mL of 70% (v/v) methanol was added. The mixture was mixed in an orbital shaker for 30 min at the highest speed and room temperature. Samples were centrifuged at 5,000 rpm for 15 min at 4°C, and the supernatant was collected. The procedure was repeated two more times, and the final volume was adjusted to 5 mL with 70% (v/v) methanol and stored at -20°C until analyses for the determination of the total phenolics, flavonoids, *ortho*-diphenols, and antioxidant activity (AA) assays. The analyses were performed in triplicate in a microplate reader using 96-well microplates. All the results were presented in terms of fresh weight (FW).

#### 2.6.1 Polyphenolic contents

The polyphenolic contents, namely total phenolics, flavonoids, and *ortho*-diphenols, were determined according to spectrophotometric methodologies previously described by Monteiro et al (Monteiro et al., 2024a). For all the analyses, a total of three technical replicates of each sample were performed and specific calibration curves were made (r^2^ total phenolics = 0.9984; r^2^ flavonoids = 0.9924; r^2^*ortho*-diphenols = 0.9993). The total phenolic and *ortho*-diphenol contents were expressed in mg of gallic acid equivalents per g of fresh weight (mg GAE g^−1^ of FW), while the flavonoid content was expressed as mg of catechin equivalents per g of fresh weight (mg CAE g^−1^ of FW).

#### 2.6.2 Antioxidant activity assays

The antioxidant activity was determined using the free radicals ABTS•+ (2,2′-azino-bis (3-ethylbenzothiazoline-6-sulphonic acid)), DPPH (2,2-diphenyl-1-picrylhydrazyl), and Ferric Reducing Antioxidant Power (FRAP) assay. These three assays were spectrophotometric methods adapted to a microscale, following the methods previously described by Monteiro et al (Monteiro et al., 2024a). Measurements were performed in triplicate, and specific calibration curves were prepared for each assay (r^2^ ABTS = 0.9927; r^2^ DPPH = 0.9779; r^2^ FRAP = 0.9943). A ninety-six-well microplate reader was used for the analysis. The results were expressed as mg of Trolox per g of fresh weight (mg Trolox g^−1^ of FW).

### 2.7 Statistical analysis

Data was analyzed using SPSS Statistics for Windows (IBM SPSS Statistics for Windows, Version 23.0, IBM Corp., Armonk, NY, USA). Statistical differences between varieties in each phenological stage of each year were evaluated by one-, two-, and three-way ANOVA, followed by Tukey multiple range test (*p<* 0.05). The results were presented as the mean with the respective standard error (SE) using GraphPad Prism version 9.0.0.12 for Windows, GraphPad Software, LLC. Correlation analysis (Pearson’s coefficient) was performed to understand how each physiological and biochemical parameter was influenced by variety, year and physiological stage. Principal component analysis (PCA) was carried out to highlight the physiological and biochemical parameters and to discriminate the grapevines distribution, using values normalized into percentages considering the maximum value obtained for each assay/test. Correlation and PCA analyses were performed in GraphPad Prism version 9.0.0.12 for Windows, GraphPad Software, LLC.

## 3 Results

### 3.1 Gas exchange measurements

The leaf gas exchange results at morning and midday, in veraison and maturity stages, are presented in **Table 1**.

**Table 1:** Leaf gas exchange measurements in twelve different varieties (V) of *Vitis vinifera* L. in the phenological stages (PS) of veraison and maturity in the years (Y) 2023 and 2024 in the morning and at midday; *E* - transpiration rate (mmol m^-2^s^-1^); *g_s_* - stomatal conductance (mmol m^-2^s^-1^); *C_i_* - intercellular CO_2_ concentration (µmol mol^-1^); *A* - net CO_2_ assimilation rate (µmol m^-2^s^-1^); *A/g_s_* - intrinsic water use efficiency (µmol mol^-1^); Values are represented as mean ± SE; Different letters and numbers indicate significant differences (*p<* 0.05, Tukey’s test) among varieties within each phenological stage (veraison – 1; maturity – 2) and year (lowercase – 2023; uppercase – 2024); Statistical analyses were performed separately for each time of day (morning and midday); ns, not significant.

Y	PS	Period	Variety	*E*	*g_s_*	*C_i_*	*A*	*A/g_s_*
2023	Veraison	Morning	‘Touriga Nacional’	2.69 ± 0.57 ab_1_	115.27 ± 17.48 ab_1_	265.74 ± 6.01 a_1_	8.47 ± 0.84 b-d_1_	74.55 ± 3.82 b_1_
			‘Touriga Franca’	2.50 ± 0.44 ab_1_	109.18 ± 19.96 a-c_1_	288.64 ± 19.68 ab_1_	6.43 ± 0.98 a-c_1_	61.50 ± 9.39 ab_1_
			‘Tinta Roriz’	3.88 ± 0.62 ab_1_	181.50 ± 33.47 bc_1_	277.61 ± 6.36 ab_1_	11.36 ± 1.37 d_1_	64.64 ± 5.86 ab_1_
			‘Tinto Cão’	3.70 ± 0.41 ab_1_	135.46 ± 13.89 a-c_1_	269.75± 11.43 a_1_	8.93 ± 0.99 b-d_1_	66.73 ± 6.63 ab_1_
			‘Tinta Barroca’	4.29 ± 0.49 b_1_	195.96 ± 20.31 c_1_	275.46 ± 2.91 a_1_	11.82 ± 0.89 d_1_	60.88 ± 2.49 ab_1_
			‘Trincadeira’	3.29 ± 0.36 ab_1_	117.22 ± 10.27 a-c_1_	271.24 ± 12.45 a_1_	8.15 ± 0.21 b-d_1_	70.33 ± 4.77 b_1_
			‘Vinhão’	3.81 ± 0.24 ab_1_	139.85 ± 20.77 a-c_1_	253.64 ± 15.35 a_1_	9.97 ± 0.78 cd_1_	74.66 ± 8.64 b_1_
			‘Donzelinho Tinto’	2.87 ± 0.30 ab_1_	86.30 ± 14.76 ab_1_	296.09 ± 27.87 ab_1_	4.39 ± 0.77 ab_1_	56.12 ± 15.50 ab_1_
			‘Alicante Bouschet’	4.53 ± 0.38 b_1_	184.72 ± 21.83 bc_1_	263.82 ± 4.32 a_1_	11.99 ± 1.03 d_1_	65.77 ± 2.73 ab_1_
			‘Touriga Fêmea’	3.08 ± 0.49 ab_1_	95.75 ± 24.92 ab_1_	288.74 ± 8.66 ab_1_	5.36 ± 0.93 a-c_1_	58.89 ± 5.82 ab_1_
			‘Malvasia Preta’	3.90 ± 0.21 ab_1_	125.09 ± 10.82 a-c_1_	249.16 ± 6.22 a_1_	9.65 ± 1.03 cd_1_	76.99 ± 2.88 a_1_
			‘Mourisco de Semente’	1.93 ± 0.07 a_1_	57.71 ± 4.42 a_1_	341.96 ± 16.96 b_1_	1.93 ± 0.67 a_1_	32.32 ± 10.49 b_1_
		Midday	‘Touriga Nacional’	4.75 ± 1.14 ns	95.40 ± 21.30 ab_1_	159.46 ± 57.62 ns	10.40 ± 2.11 ns	118.26 ± 28.70 ab_1_
			‘Touriga Franca’	3.80 ± 1.05 ns	74.33 ± 32.03 ab_1_	244.41 ± 25.09 ns	6.11 ± 3.22 ns	77.86 ± 13.14 ab_1_
			‘Tinta Roriz’	7.14 ± 1.45 ns	160.85 ± 46.63 b_1_	264.93 ± 31.09 ns	10.62 ± 4.03 ns	60.62 ± 15.25 ab_1_
			‘Tinto Cão’	2.75 ± 0.10 ns	48.88 ± 4.30 ab_1_	117.65 ± 48.10 ns	7.60 ± 1.83 ns	152.12 ± 23.47 b_1_
			‘Tinta Barroca’	5.87 ± 0.62 ns	115.65 ± 12.08 ab_1_	176.29 ± 45.40 ns	12.11 ± 1.67 ns	108.98 ± 22.53 ab_1_
			‘Trincadeira’	4.87 ± 0.44 ns	97.63 ± 10.80 ab_1_	202.50 ± 37.39 ns	9.44 ± 1.58 ns	98.34 ± 19.02 ab_1_
			‘Vinhão’	4.13 ± 0.85 ns	77.25 ± 20.83 ab_1_	154.31 ± 26.45 ns	9.54 ± 2.04 ns	127.67 ± 15.00 ab_1_
			‘Donzelinho Tinto’	4.09 ± 1.35 ns	66.01 ± 24.38 ab_1_	214.61 ± 59.16 ns	4.83 ± 0.33 ns	98.72 ± 36.17 ab_1_
			‘Alicante Bouschet’	6.16 ± 0.43 ns	118.64 ± 11.21 ab_1_	225.99 ± 42.500 ns	10.08 ± 2.96 ns	83.25 ± 22.33 ab_1_
			‘Touriga Fêmea’	2.71 ± 0.97 ns	47.27 ± 20.06 a_1_	175.28 ± 17.38 ns	5.89 ± 2.52 ns	120.30 ± 8.78 ab_1_
			‘Malvasia Preta’	3.22 ± 0.76 ns	49.27 ± 11.93 ab_1_	244.10 ± 28.58 ns	4.18 ± 1.34 ns	81.78 ± 16.11 ab_1_
			‘Mourisco de Semente’	4.28 ± 1.04 ns	61.17 ± 15.97 ab_1_	312.12 ± 16.62 ns	2.74 ± 1.21 ns	36.76 ± 10.15 a_1_
	Maturity	Morning	‘Touriga Nacional’	2.30 ± 0.37 ns	104.11 ± 13.26 a-c_2_	289.04 ± 16.46 ab_2_	5.94 ± 0.97 ab_2_	58.92 ± 9.26 ab_2_
			‘Touriga Franca’	2.74 ± 0.21 ns	129.93 ± 10.57 c_2_	287.81 ± 10.50 ab_2_	7.61 ± 1.21 b_2_	57.89 ± 5.38 ab_2_
			‘Tinta Roriz’	2.39 ± 0.29 ns	104.58 ± 11.87 a-c_2_	253.69 ± 8.58 ab_2_	8.03 ± 0.37 b_2_	77.96 ± 5.32 ab_2_
			‘Tinto Cão’	3.16 ± 0.12 ns	128.42 ± 5.13 c_2_	299.76 ± 11.34 ab_2_	6.64 ± 0.66 b_2_	51.99 ± 5.88 ab_2_
			‘Tinta Barroca’	2.74 ± 0.30 ns	107.95 ± 4.98 a-c_2_	265.25 ± 9.53 ab_2_	7.70 ± 0.84 b_2_	70.76 ± 4.48 ab_2_
			‘Trincadeira’	3.03 ± 0.42 ns	119.80 ± 11.68 bc_2_	292.94 ± 2.89 ab_2_	6.59 ± 0.43 b_2_	55.58 ± 2.47 ab_2_
			‘Vinhão’	2.90 ± 0.26 ns	111.23 ± 8.22 bc_2_	261.55 ± 10.83 ab_2_	7.95 ± 0.56 b_2_	72.55 ± 6.83 ab_2_
			‘Donzelinho Tinto’	2.00 ± 0.32 ns	68.06 ± 8.76 a-c_2_	258.52 ± 16.66 ab_2_	5.45 ± 1.22 ab_2_	78.23 ± 8.21 ab_2_
			‘Alicante Bouschet’	2.75 ± 0.63 ns	102.75 ± 23.46 a-c_2_	258.88 ± 11.10 ab_2_	7.31 ± 1.247 b_2_	75.83 ± 7.94 ab_2_
			‘Touriga Fêmea’	1.89 ± 0.23 ns	62.41 ± 11.72 ab_2_	295.56 ± 20.94 ab_2_	3.75 ± 1.35 ab_2_	57.90 ± 11.61 ab_2_
			‘Malvasia Preta’	2.49 ± 0.36 ns	90.76 ± 9.81 a-c_2_	234.52 ± 7.95 a_2_	8.04 ± 0.37 b_2_	89.81 ± 5.83 a_2_
			‘Mourisco de Semente’	1.47 ± 0.42 ns	43.38 ± 15.36 a_2_	318.73 ± 22.51 b_2_	1.82 ± 0.65 a_2_	44.50 ± 13.76 b_2_
		Midday	‘Touriga Nacional’	3.01 ± 0.16 a-c_2_	68.23 ± 5.22 ab_2_	200.87 ± 19.46 ab_2_	7.293 ± 0.91 ns	106.85 ± 11.35 ab_2_
			‘Touriga Franca’	1.64 ± 0.19 ab_2_	39.17 ± 2.87 ab_2_	112.86 ± 35.30 a_2_	6.43 ± 0.93 ns	164.50 ± 21.08 b_2_
			‘Tinta Roriz’	3.71 ± 0.76 a-c_2_	93.84 ± 21.46 b_2_	213.37 ± 29.20 ab_2_	8.28 ± 1.45 ns	97.25 ± 16.77 ab_2_
			‘Tinto Cão’	3.22 ± 0.38 a-c_2_	76.82 ± 7.82 ab_2_	196.53 ± 21.72 ab_2_	8.14 ± 0.92 ns	107.27 ± 11.93 ab_2_
			‘Tinta Barroca’	3.60 ± 0.32 a-c_2_	77.79 ± 4.74 ab_2_	242.84 ± 38.18 ab_2_	6.21 ± 1.54 ns	81.78 ± 21.86 ab_2_
			‘Trincadeira’	3.22 ± 0.27 a-c_2_	74.74 ± 4.88 ab_2_	221.36 ± 33.50 ab_2_	6.85 ± 0.95 ns	93.87 ± 18.14 ab_2_
			‘Vinhão’	4.11 ± 0.47 bc_2_	94.49 ± 5.36 b_2_	229.64 ± 35.90 ab_2_	7.07 ± 1.60 ns	87.28 ± 19.79 ab_2_
			‘Donzelinho Tinto’	4.40 ± 0.51 c_2_	101.47 ± 14.87 b_2_	238.15 ± 24.92 ab_2_	7.71 ± 0.35 ns	82.61 ± 15.536 ab_2_
			‘Alicante Bouschet’	4.00 ± 0.60 a-c_2_	92.70 ± 15.21 b_2_	219.26 ± 22.99 ab_2_	9.43 ± 1.40 ns	93.59 ± 13.94 ab_2_
			‘Touriga Fêmea’	2.86 ± 0.48 a-c_2_	65.74 ± 16.43 ab_2_	214.80 ± 12.75 ab_2_	6.64 ± 1.93 ns	98.07 ± 6.13 ab_2_
			‘Malvasia Preta’	1.57 ± 0.33 a_2_	27.86 ± 5.07 a_2_	141.91 ± 56.65 ab_2_	3.75 ± 0.29 ns	147.46 ± 35.98 ab_2_
			‘Mourisco de Semente’	2.75 ± 0.53 a-c_2_	51.68 ± 11.89 ab_2_	289.47 ± 20.15 b_2_	3.11 ± 1.21 ns	54.72 ± 11.93 a_2_
2024	Veraison	Morning	‘Touriga Nacional’	3.02 ± 1.15 ns	113.954 ± 27.38 ns	277.32 ± 3.89 ns	8.04 ± 1.45 AB_1_	73.71 ± 6.55 ns
			‘Touriga Franca’	3.83 ± 0.46 ns	138.97 ± 35.30 ns	263.45 ± 5.75 ns	10.30 ± 2.286 B_1_	75.68 ± 4.65 ns
			‘Tinta Roriz’	4.36 ± 0.76 ns	142.59 ± 10.11 ns	278.97 ± 14.70 ns	9.53 ± 0.95 B_1_	67.56 ± 8.32 ns
			‘Tinto Cão’	1.88 ± 0.44 ns	45.13 ± 4.19 ns	293.61 ± 12.01 ns	3.00 ± 0.11 A_1_	68.23 ± 9.31 ns
			‘Tinta Barroca’	2.74 ± 0.18 ns	90.01 ± 18.46 ns	253.96 ± 9.25 ns	7.47 ± 1.09 AB_1_	86.72 ± 6.17 ns
			‘Trincadeira’	3.16 ± 0.35 ns	103.12 ± 19.64 ns	245.38 ± 14.30 ns	9.33 ± 2.37 B_1_	88.30 ± 5.59 ns
			‘Vinhão’	2.10 ± 0.57 ns	49.37 ± 8.35 ns	243.80 ± 4.73 ns	4.72 ± 0.71 AB_1_	96.20 ± 2.66 ns
			‘Donzelinho Tinto’	2.85 ± 0.23 ns	90.85 ± 30.54 ns	253.60 ± 26.88 ns	6.97 ± 0.70 AB_1_	87.61 ± 16.30 ns
			‘Alicante Bouschet’	2.69 ± 0.99 ns	64.24 ± 20.53 ns	241.13 ± 26.24 ns	5.58 ± 0.92 AB_1_	97.28 ± 18.62 ns
			‘Touriga Fêmea’	2.68 ± 0.23 ns	59.29 ± 5.33 ns	265.71 ± 17.31 ns	4.83 ± 0.98 AB_1_	79.77 ± 10.92 ns
			‘Malvasia Preta’	3.65 ± 0.73 ns	87.20 ± 14.30 ns	246.38 ± 20.85 ns	7.50 ± 0.11 AB_1_	90.52 ± 13.96 ns
			‘Mourisco de Semente’	2.87 ± 0.55 ns	66.95 ± 12.42 ns	271.16 ± 17.81 ns	5.04 ± 0.70 AB_1_	77.76 ± 9.79 ns
		Midday	‘Touriga Nacional’	2.35 ± 0.66 ns	57.91 ± 17.59 ns	201.42 ± 1.93 ns	6.64 ± 1.89 AB_1_	116.63 ± 3.74 ns
			‘Touriga Franca’	4.00 ± 0.43 ns	128.54 ± 22.74 ns	262.27 ± 11.24 ns	9.75 ± 2.20 AB_1_	74.72 ± 3.45 ns
			‘Tinta Roriz’	3.08 ± 0.83 ns	81.46 ± 31.31 ns	191.74 ± 29.64 ns	8.47 ± 1.50 AB_1_	119.83 ± 20.67 ns
			‘Tinto Cão’	3.51 ± 0.80 ns	102.70 ± 31.53 ns	278.61 ± 7.73 ns	7.08 ± 2.25 AB_1_	67.99 ± 1.83 ns
			‘Tinta Barroca’	2.15 ± 0.52 ns	50.36 ± 14.81 ns	245.29 ± 24.41 ns	4.29 ± 0.70 A_1_	92.30 ± 15.78 ns
			‘Trincadeira’	4.16 ± 0.56 ns	135.10 ± 27.69 ns	267.13 ± 13.92 ns	9.44 ± 1.50 AB_1_	72.88 ± 9.15 ns
			‘Vinhão’	2.45 ± 0.88 ns	55.51 ± 21.40 ns	215.73 ± 48.93 ns	4.83 ± 0.74 AB_1_	110.51 ± 32.65 ns
			‘Donzelinho Tinto’	4.15 ± 0.77 ns	146.25 ± 34.81 ns	190.92 ± 41.84 ns	13.84 ± 0.18 B_1_	109.01 ± 25.95 ns
			‘Alicante Bouschet’	2.88 ± 0.26 ns	77.09 ± 11.06 ns	203.40 ± 34.906 ns	8.98 ± 2.60 AB_1_	111.62 ± 17.39 ns
			‘Touriga Fêmea’	2.63 ± 0.12 ns	61.87 ± 3.24 ns	192.51 ± 46.36 ns	7.30 ± 1.18 AB_1_	120.77 ± 26.83 ns
			‘Malvasia Preta’	2.16 ± 0.17 ns	50.70 ± 3.55 ns	222.96 ± 4.85 ns	5.35 ± 0.47 AB_1_	105.30 ± 2.02 ns
			‘Mourisco de Semente’	2.25 ± 0.44 ns	58.48 ± 16.76 ns	204.42 ± 25.37 ns	7.15 ± 2.56 AB_1_	112.63 ± 13.02 ns
	Maturity	Morning	‘Touriga Nacional’	1.63 ± 0.47 ns	69.14 ± 13.17 A_2_	238.68 ± 11.87 ns	6.28 ± 0.75 AB_2_	94.93 ± 8.27 ns
			‘Touriga Franca’	2.80 ± 0.44 ns	124.42 ± 16.40 B_2_	256.43 ± 1.35 ns	9.64 ± 1.11 B_2_	77.86 ± 1.64 ns
			‘Tinta Roriz’	2.03 ± 0.31 ns	82.31 ± 13.63 AB_2_	233.89 ± 12.06 ns	7.61 ± 0.84 AB_2_	95.01 ± 8.61 ns
			‘Tinto Cão’	1.84 ± 0.20 ns	93.07 ± 9.07 AB_2_	268.823 ± 7.38 ns	7.09 ± 0.94 AB_2_	75.63 ± 2.77 ns
			‘Tinta Barroca’	2.18 ± 0.39 ns	90.46 ± 2.05 AB_2_	238.01 ± 24.82 ns	8.26 ± 1.08 AB_2_	91.85 ± 13.50 ns
			‘Trincadeira’	1.18 ± 0.16 ns	52.77 ± 8.21 A_2_	240.08 ± 31.11 ns	4.82 ± 0.32 A_2_	97.35 ± 19.21 ns
			‘Vinhão’	1.20 ± 0.29 ns	53.14 ± 9.45 A_2_	243.11 ± 9.09 ns	5.03 ± 0.88 A_2_	95.02 ± 4.99 ns
			‘Donzelinho Tinto’	1.57 ± 0.27 ns	75.27 ± 5.71 AB_2_	216.38 ± 8.85 ns	8.04 ± 0.85 AB_2_	106.31 ± 3.58 ns
			‘Alicante Bouschet’	1.47 ± 0.31 ns	56.60 ± 4.98 A_2_	217.63 ± 10.52 ns	6.11 ± 0.54 AB_2_	108.31 ± 6.30 ns
			‘Touriga Fêmea’	1.45 ± 0.05 ns	69.83 ± 9.75 A_2_	237.62 ± 8.96 ns	6.54 ± 0.56 AB_2_	95.25 ± 5.76 ns
			‘Malvasia Preta’	2.17 ± 0.41 ns	84.79 ± 8.83 AB_2_	238.09 ± 8.37 ns	7.73 ± 0.32 AB_2_	92.28 ± 5.66 ns
			‘Mourisco de Semente’	1.46 ± 0.32 ns	57.44 ± 7.84 A_2_	274.10 ± 23.70 ns	4.58 ± 1.27 A_2_	75.54 ± 13.02 ns
		Midday	‘Touriga Nacional’	2.23 ± 0.55 AB_2_	69.36 ± 15.49 AB_2_	234.20 ± 22.78 AB_2_	6.00 ± 0.60 A-C_2_	92.43 ± 13.16 AB_2_
			‘Touriga Franca’	3.49 ± 0.54 B_2_	122.51 ± 13.65 B_2_	269.51 ± 26.60 AB_2_	8.26 ± 1.56 BC_2_	67.80 ± 12.76 A_2_
			‘Tinta Roriz’	2.66 ± 0.41 AB_2_	83.00 ± 13.11 AB_2_	228.03 ± 24.11 AB_2_	7.32 ± 0.48 A-C_2_	94.96 ± 14.47 AB_2_
			‘Tinto Cão’	2.37 ± 0.18 AB_2_	62.18 ± 8.61 A_2_	240.77 ± 15.80 AB_2_	5.48 ± 0.49 A-C_2_	89.96 ± 9.44 AB_2_
			‘Tinta Barroca’	1.68 ± 0.10 AB_2_	45.38 ± 3.59 A_2_	172.82 ± 18.79 A_2_	6.00 ± 0.84 A-C_2_	131.20 ± 9.963 B_2_
			‘Trincadeira’	2.39 ± 0.68 AB_2_	67.98 ± 21.63 AB_2_	280.56 ± 33.16 B_2_	3.86 ± 0.81 AB_2_	68.54 ± 20.86 A_2_
			‘Vinhão’	2.54 ± 0.23 AB_2_	67.23 ± 5.48 AB_2_	242.49 ± 4.25 AB_2_	5.90 ± 0.43 A-C_2_	87.86 ± 1.94 AB_2_
			‘Donzelinho Tinto’	2.77 ± 0.13 AB_2_	75.96 ± 9.65 AB_2_	206.23 ± 9.83 AB_2_	8.12 ± 1.10 BC_2_	107.14 ± 4.73 AB_2_
			‘Alicante Bouschet’	1.92 ± 0.20 AB_2_	48.44 ± 2.90 A_2_	215.17 ± 19.91 AB_2_	5.04 ± 0.28 A-C_2_	105.59 ± 12.14 AB_2_
			‘Touriga Fêmea’	2.37 ± 0.11 AB_2_	66.91 ± 7.39 AB_2_	232.17 ± 16.59 AB_2_	6.33 ± 0.95 A-C_2_	94.30 ± 9.70 AB_2_
			‘Malvasia Preta’	3.01 ± 0.41 AB_2_	80.12 ± 14.95 AB_2_	198.81 ± 13.55 AB_2_	8.68 ± 1.28 C_2_	110.35 ± 8.23 AB_2_
			‘Mourisco de Semente’	1.44 ± 0.17 A_2_	33.93 ± 6.96 A_2_	225.66 ± 5.32 AB_2_	3.43 ± 0.70 A_2_	101.13 ± 3.10 AB_2_
Morning			*p* value (V)	0.060	<0.001	<0.001	<0.001	<0.001
			*p* value (PS)	<0.001	0.001	0.060	0.039	0.058
			*p* value (Y)	<0.001	<0.001	<0.001	0.066	<0.001
			*p* value (V × PS)	0.082	0.059	0.189	0.153	0.103
			*p* value (V × Y)	0.061	0.001	0.183	<0.001	0.314
			*p* value (Y × PS)	0.350	0.095	0.250	0.072	0.471
			*p* value (V × PS × Y)	<0.001	<0.001	0.017	<0.001	<0.001
Midday			*p* value (V)	0.013	<0.001	0.265	0.006	0.241
			*p* value (PS)	0.002	0.280	0.494	0.083	0.681
			*p* value (Y)	<0.001	0.703	0.071	0.491	0.713
			*p* value (V × PS)	0.320	0.226	0.346	0.320	0.324
			*p* value (V × Y)	0.001	<0.001	0.014	0.012	0.009
			*p* value (Y × PS)	0.116	0.947	0.807	0.779	0.385
			*p* value (V × PS × Y)	<0.001	<0.001	0.003	0.013	0.002

Transpiration rate (*E*) varied from 1.18 mmol m^-2^s^-1^ in ‘Trincadeira’ (maturity, 2024) to 4.53 mmol m^-2^s^-1^ in ‘Alicante Bouschet’ (veraison, 2023) in the morning and 1.44 mmol m^-2^s^-1^ in ‘Mourisco de Semente’ (maturity, 2024) to 7.14 mmol m^-2^s^-1^ in ‘Tinta Roriz’ (veraison, 2023) in midday. In the morning period, significant differences (**Table 1**) were observed for year (*p<* 0.001) and phenological stage (*p<* 0.001) and by the interaction of year × phenological stage × variety (*p<* 0.001), but not for variety. During the midday period, significant differences (**Table 2**) were detected for year (*p<* 0.001), phenological stage (*p<* 0.01), variety (*p<* 0.05), for the interaction variety × year (*p<* 0.01), and for the interaction variety × phenological stage × year (*p<* 0.001). *E* decreased from veraison to maturity in both years in all varieties except for ‘Touriga Franca’ in 2023. Significant differences between varieties were found in the morning results in veraison of 2023, and in the midday results in maturity of both years.

**Table 2:** Total leaf soluble sugars in twelve different varieties of *Vitis vinifera* in the phenological stages of veraison and maturity in the years 2023 and 2024.

Variety	Leaf soluble sugars (mg g^-1^ FW)			
	Veraison 2023	Maturity 2023	Veraison 2024	Maturity 2024
‘Touriga Nacional’	29.65 ± 0.57 ns	36.37± 1.18 b-d_2_	16.35 ± 0.80 A_1_	33.15 ± 2.32 A-C_2_
‘Touriga Franca’	37.17 ± 2.92 ns	32.98 ± 1.16 a-d_2_	22.56 ± 2.26 AB_1_	29.04 ± 1.48 AB_2_
‘Tinta Roriz’	34.02 ± 0.53 ns	32.16 ± 1.16 a-c_2_	21.26 ± 1.51 AB_1_	28.90 ± 1.74 AB_2_
‘Tinto Cão’	27.03 ± 2.44 ns	27.02 ± 1.36 a_2_	26.43 ± 1.10 BC_1_	28.38 ± 1.77 AB_2_
‘Tinta Barroca’	27.92 ± 2.20 ns	33.61 ± 2.31 a-d_2_	22.39 ± 1.10 AB_1_	30.28 ± 2.21 AB_2_
‘Trincadeira’	29.17 ± 1.70 ns	31.72 ± 1.69 a-c_2_	21.40 ± 1.47 AB_1_	32.91 ± 1.88 A-C_2_
‘Vinhão’	24.81 ± 1.54 ns	29.12 ± 2.45 ab_2_	20.98 ± 1.64 AB_1_	28.14 ± 0.44 AB_2_
‘Donzelinho Tinto’	39.65 ± 2.32 ns	35.55 ± 1.13 b-d_2_	28.19 ± 2.13 BC_1_	32.40 ± 3.22 A-C_2_
‘Alicante Bouschet’	31.18 ± 1.11 ns	33.71 ± 0.76 a-d_2_	20.77 ± 0.40 AB_1_	25.50 ± 0.35 A_2_
‘Touriga Fêmea’	45.09 ± 1.21 ns	38.11 ± 2.35 cd_2_	33.68 ± 1.23 C_1_	37.83 ± 2.19 BC_2_
‘Malvasia Preta’	38.01 ± 2.88 ns	30.95 ± 1.57 a-c_2_	25.71 ± 3.12 BC_1_	36.47 ± 2.15 BC_2_
‘Mourisco de Semente’	43.87 ± 1.33 ns	40.51 ± 1.72 d_2_	21.28 ± 2.66 AB_1_	42.22 ± 2.11 C_2_
*p* value (V)	<0.001			
*p* value (PS)	<0.001			
*p* value (Y)	<0.001			
*p* value (V × PS)	0.054			
*p* value (V × Y)	0.229			
*p* value (Y × PS)	<0.001			
*p* value (V × PS × Y)	<0.001			

Values are represented as mean ± SE and the different letters and numbers represent significant differences (*p<* 0.05, Tukey’s test) between the twelve different varieties within each phenological stage (veraison – 1; maturity – 2) and each year (lowercase – 2023; uppercase – 2024).

Stomatal conductance (*g_s_*) varied between 43.38 mmol m^-2^s^-1^ in ‘Mourisco de Semente’ (maturity, 2024) and 195.96 mmol m^-2^s^-1^ in ‘Tinta Barroca’ (veraison, 2023) in the morning and 27.86 mmol m^-2^s^-1^ in ‘Malvasia Preta’ (maturity, 2023) and 160.85 mmol m^-2^s^-1^ in ‘Tinta Roriz’ (veraison, 2023) at midday. In the morning, *g_s_* was significantly influenced (**Table 1**) by the year (*p<* 0.001), the phenological stage (*p<* 0.01), the variety (*p<* 0.001), and by the interactions variety × year (*p<* 0.01) and variety × phenological stage × year (*p<* 0.001). At midday, significant effects were observed only for the variety (*p<* 0.001) and the interactions variety × year (*p<* 0.001) and variety × phenological stage × year (*p<* 0.001). In general, *g_s_* decreased from veraison to maturity and from morning to midday. The only varieties that behaved differently were ‘Touriga Franca’ and ‘Trincadeira’ which suffered an increase of *g_s_* from veraison to maturity in 2023 and ‘Tinto Cão’, ‘Vinhão’, and ‘Touriga Fêmea’ in 2024. Significant statistical differences were found between varieties in the two years and phenological stages, in both morning and midday periods, apart from veraison 2024 in either period.

Intercellular CO_2_ concentration (*C_i_*) varied between 216.38 µmol mol^-1^ in ‘Donzelinho Tinto’ (maturity, 2024) and 341.96 µmol mol^-1^ in ‘Mourisco de Semente’ (veraison, 2023) in the morning and 112.86 µmol mol^-1^ in ‘Touriga Franca’ (maturity, 2023) and 312.12 µmol mol^-1^ in ‘Mourisco de Semente’ (veraison, 2023) at midday. *C_i_* showed interference of the year (*p<* 0.001) and the variety (*p<* 0.001) in the morning period, of the interaction variety × year (*p<* 0.05) at midday and of the interaction variety × phenological stage × year (*p<* 0.05) in both day periods (**Table 1**). In general, *C_i_* decreased from veraison to maturity in both years and from morning measurements to midday measurements. In 2023, ‘Touriga Nacional’, ‘Tinto Cão’, ‘Trincadeira’, Vinhão’, and ‘Touriga Fêmea’ and in 2024 ‘Mourisco de Semente’ did not follow the pattern of the other varieties, therefore, increasing *C_i_* from veraison to maturity. Significant statistical differences between varieties at both phenological stages were found in the morning in the year 2023 and in maturity of both years at midday.

Net CO_2_ assimilation rate (*A*) varied between 1.82 µmol m^-2^s^-1^ in ‘Mourisco de Semente’ (maturity, 2023) and 11.99 µmol m^-2^s^-1^ in ‘Alicante Bouschet’ (veraison, 2023) in the morning and 2.74 µmol m^-2^s^-1^ in ‘Mourisco de Semente’ (veraison, 2023) and 13.84 µmol m^-2^s^-1^ in ‘Donzelinho Tinto’ (veraison, 2024) at midday. *A* was influenced by the phenological stage (*p<* 0.05), by the variety (*p<* 0.001), and by the interactions variety × year (*p<* 0.001) and variety × phenological stage × year (*p<* 0.001) in the morning. At midday, results were influenced by the variety (*p<* 0.01), and by the interactions variety × year (*p<* 0.05), and variety × phenological stage × year (*p<* 0.01) (**Table 1**). *A* decreased from veraison to maturity in all varieties in 2023 except for ‘Touriga Franca’ and ‘Donzelinho Tinto’. In 2024, there was an increase of *A* in seven of the twelve varieties. In general, there was a decrease in *A* from the morning to midday in both years. In the morning significant statistical differences were found among varieties, in both years and phenological stages. In the midday measurements, significant statistical differences (**Table 1**) were only found between varieties in both phenological stages in the year 2024.

Intrinsic water use efficiency (*A/g_s_*) varied between 32.32 µmol mol^-1^ in ‘Mourisco de Semente’ (veraison, 2023) and 108.31 µmol mol^-1^ in ‘Alicante Bouschet’ (maturity, 2024) in the morning and 36.76 µmol mol^-1^ in ‘Mourisco de Semente’ (veraison, 2023) and 164.50 µmol mol^-1^ in ‘Touriga Franca’ (maturity, 2023) at midday. *A/g_s_* was influenced at the morning by the year (*p<* 0.001) and by the variety (*p<* 0.001), at midday by the interaction variety × year (*p<* 0.01) and both daily measurements were influenced by the interaction variety × phenological stage × year (*p<* 0.001 in the morning and *p<* 0.01 at midday). In general, *A/g_s_* increased from veraison to maturity and from morning to midday in both years. Significant statistical differences between varieties were found in the morning measurement in 2023 and at midday in both phenological stages of the year 2023 and in maturity of 2024.

### 3.2 Lipid peroxidation (TBARS)

**Figure 2:**
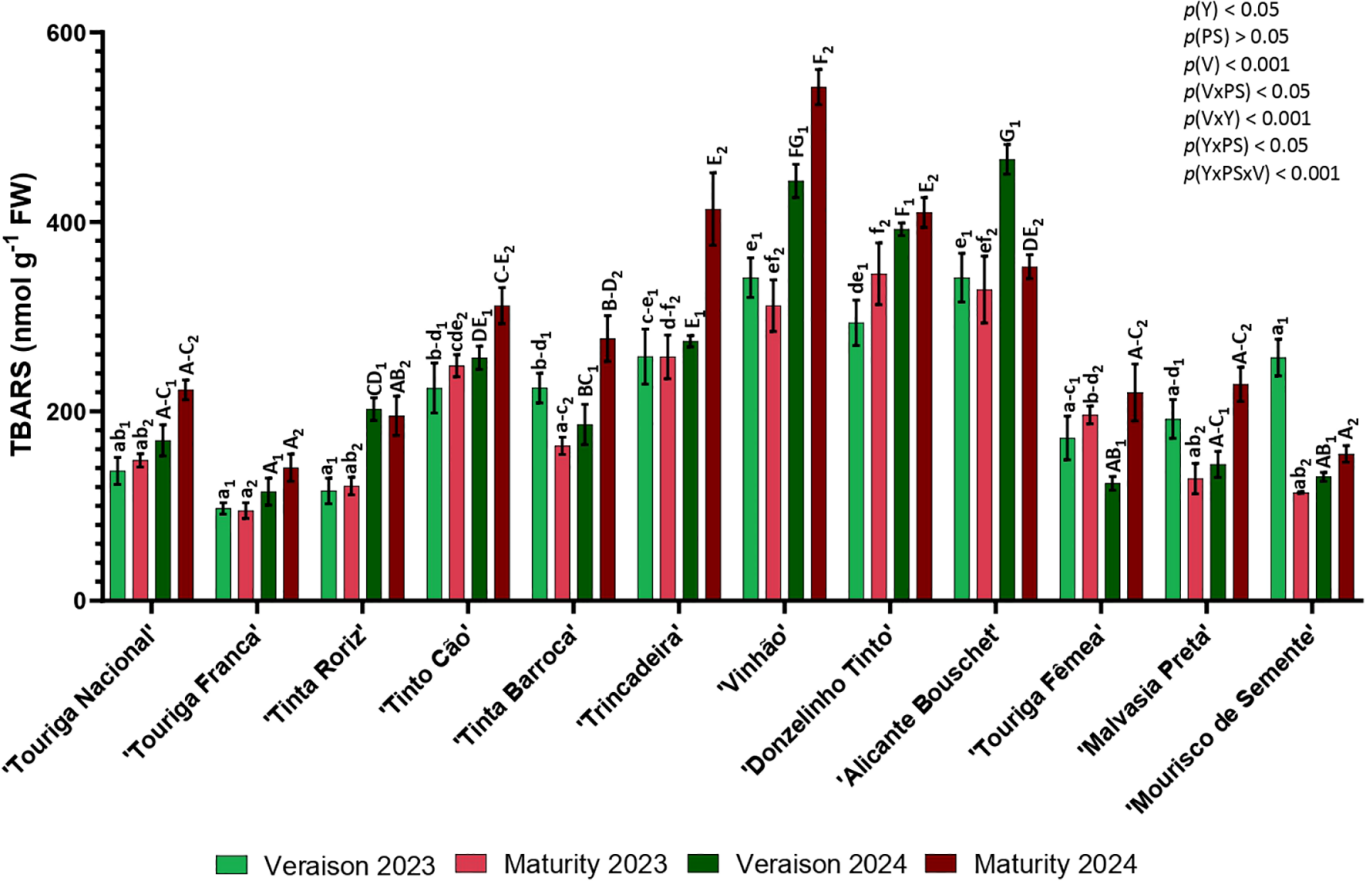
Content of TBARS in twelve different grapevine varieties (V) in two phenological stages (PS; veraison and maturity) in the years (Y) 2023 and 2024. Values are represented as mean ± SE and the different letters and numbers represent significant differences (*p<* 0.05, Tukey’s test) between the varieties within each phenological stage (veraison – 1; maturity – 2) and year (lowercase – 2023; uppercase – 2024).

The lipid peroxidation was determined using the TBARS assay (**Figure 2**). TBARS levels were higher in average in the maturity of 2024, followed by veraison 2024, veraison 2023 and maturity 2023. In general, an increase in TBARS levels was observed throughout phenological development, ranging at veraison from 97.55 nmol g^-1^ in ‘Touriga Franca’ (2023) to 466.01 nmol g^-1^ in ‘Alicante Bouschet’ (2024) and at maturity from 95.16 in ‘Touriga Franca’ (2023) to 542.26 nmol g^-1^ in ‘Vinhão’ (2024). Hence, ‘Touriga Franca’ presented the lowest content of TBARS in both phenological stages and in both years. On other hand, the varieties with highest TBARS content at veraison in both years were ‘Vinhão’ and ‘Alicante Bouschet’, while at maturity, in 2023 were ‘Donzelinho Tinto’ and ‘Alicante Bouschet’, and in 2024 ‘Vinhão’ and ‘Trincadeira’. Significant differences were observed in year (*p<* 0.05), variety (*p<* 0.001), and in the interactions variety × phenological stage (*p<* 0.05), variety × year (*p<* 0.001), year × phenological stage (*p<* 0.05) and year × phenological stage × variety (*p<* 0.001).

### 3.3 Photosynthetic pigments

**Figure 3:**
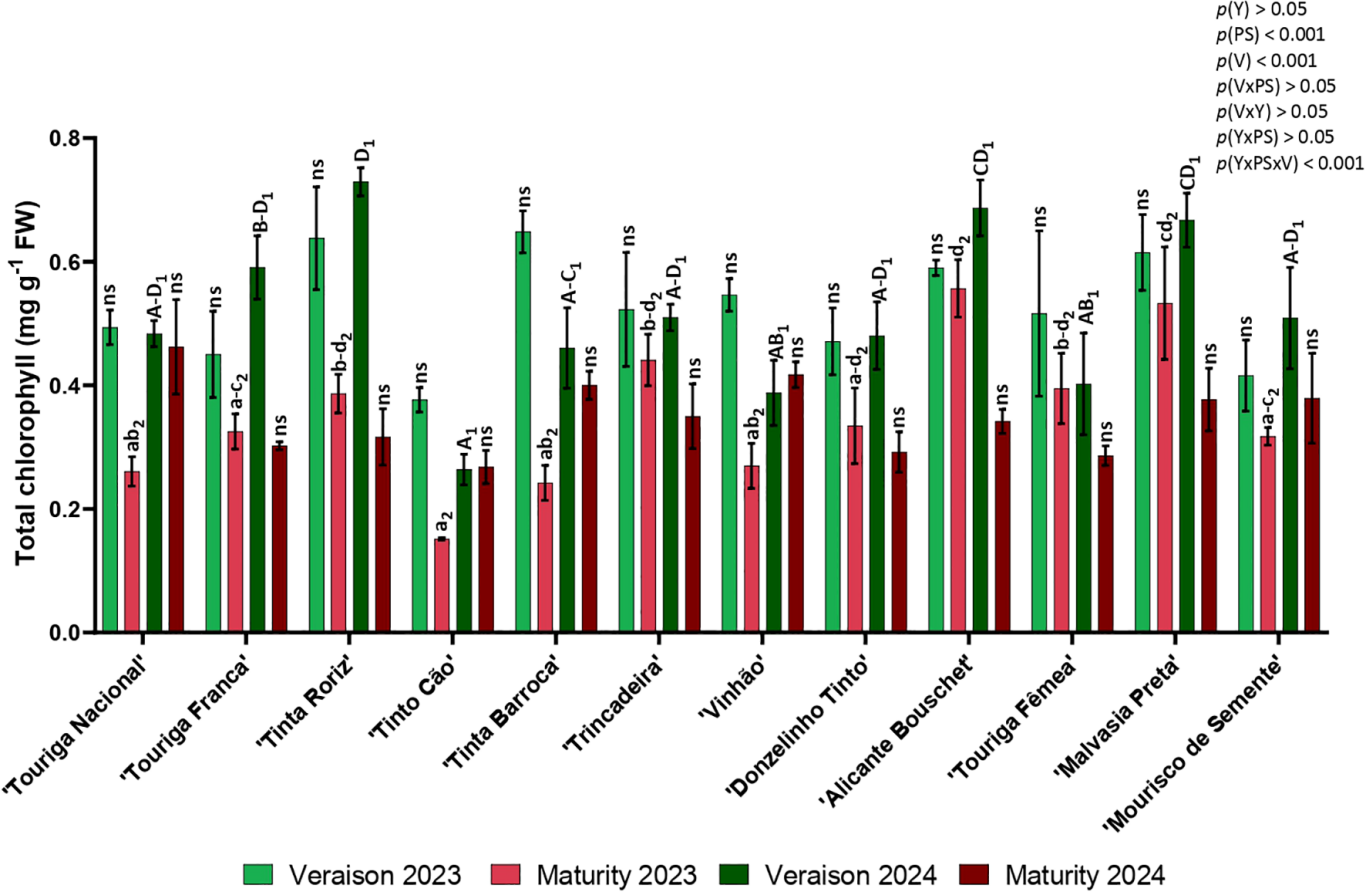
Total chlorophyll in twelve different grapevine varieties (V) in the phenological stages (PS) of veraison and maturity in the years (Y) 2023 and 2024. Values are represented as mean ± SE and the different letters and numbers represent significant differences (*p<* 0.05, Tukey’s test) between the varieties within each phenological stage (veraison – 1; maturity – 2) and year (lowercase – 2023; uppercase – 2024).

The total chlorophylls data is presented in **Figure 3**. There was a decrease in the photosynthetic pigments content from veraison to maturity in both years for all the varieties, except for ‘Tinto Cão’ and ‘Vinhão’ in 2024. Contents varied in veraison from 0.264 (‘Tinto Cão’, 2024) to 0.729 mg g^-1^ (‘Tinta Roriz’, 2024) and in maturity from 0.151 (‘Tinto Cão’, 2023) to 0.557 mg g^-1^ (‘Alicante Bouschet’, 2023). The averages value of the 12 varieties was higher at veraison 2023, followed by veraison 2024, maturity 2023 and maturity 2024. In 2023, the varieties with higher content of total chlorophyll in veraison were ‘Tinta Roriz’, ‘Tinta Barroca’, ‘Alicante Bouschet’, and ‘Malvasia Preta’, while in maturity were the varieties ‘Alicante Bouschet’ and ‘Malvasia Preta’. In 2024, the varieties with highest chlorophyll content were also ‘Tinta Roriz’, ‘Alicante Bouschet’ and ‘Malvasia Preta’ in veraison and ‘Touriga Nacional’, ‘Tinta Barroca’, and ‘Vinhão’ in maturity. ‘Tinto Cão’ was the variety with lower content of total chlorophyll in both years and in both phenological stages. Significant differences were found for variety (*p<* 0.001), phenological stage (*p<* 0.001), and in the interaction of year × phenological stage × variety (*p<* 0.001).

**Figure 4:**
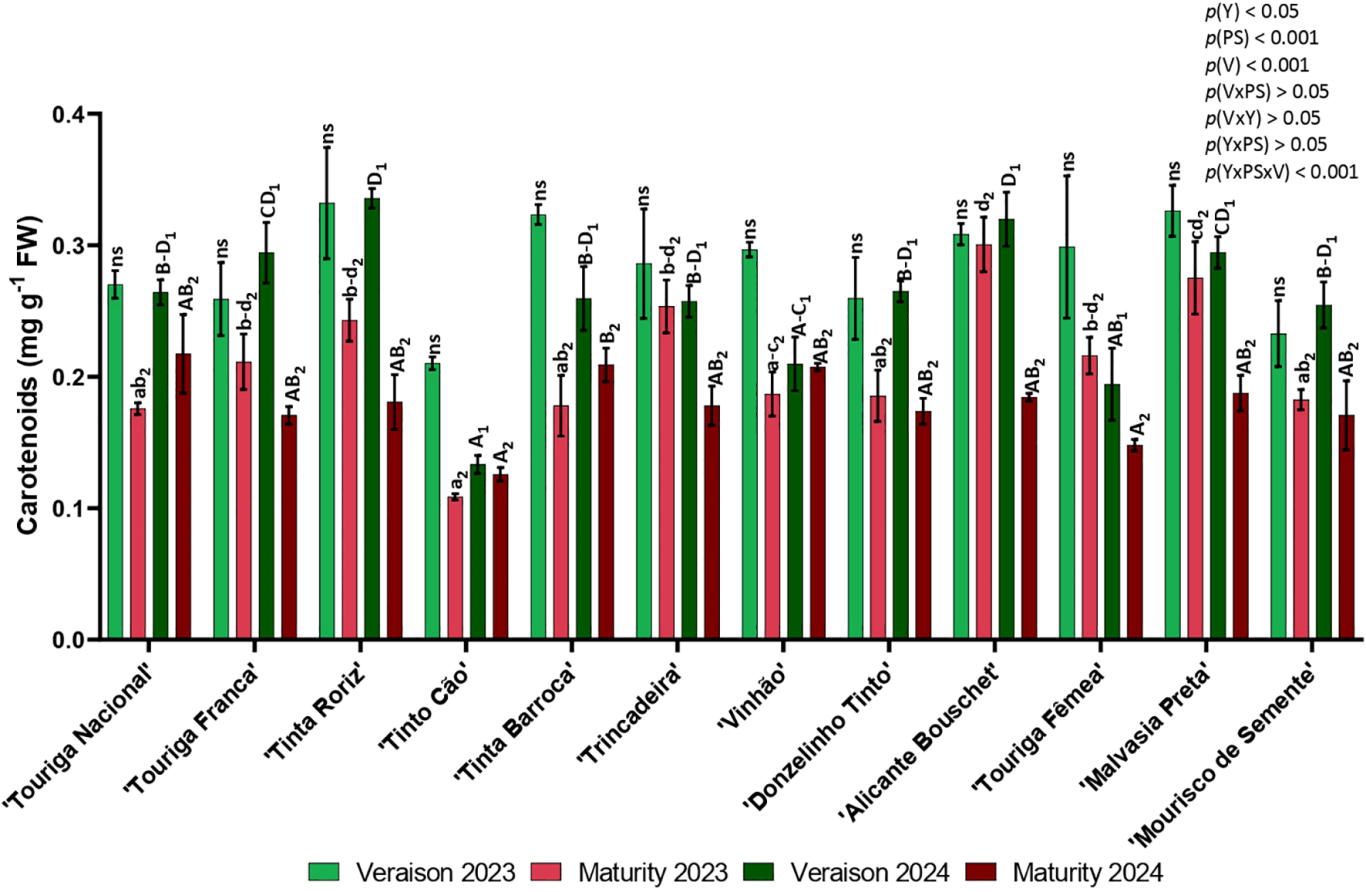
Content of carotenoids in twelve different varieties (V) of *Vitis vinifera* in the phenological stages (PS) of veraison and maturity in the years (Y) 2023 and 2024. Values are represented as mean ± SE and the different letters and numbers represent significant differences (*p<* 0.05, Tukey’s test) between the varieties within each phenological stage (veraison – 1; maturity – 2) and each year (lowercase – 2023; uppercase – 2024).

Carotenoids content decreased from veraison to maturity in all twelve varieties in both years of the study (**Figure 4**). The twelve varieties average value was higher in veraison 2023, followed by veraison 2024, maturity 2023 and finally maturity 2024. The content of carotenoids varied in veraison from 0.133 mg g^-1^ (‘Tinto Cão’, 2024) to 0.336 mg g^-1^ (‘Tinta Roriz’, 2024) and in maturity from 0.109 (‘Tinto Cão’, 2023) to 0.301 mg g^-1^ (‘Alicante Bouschet’, 2023). In 2023, ‘Tinta Roriz’, ‘Tinta Barroca’, ‘Alicante Bouschet’, and ‘Malvasia Preta’ were the ones with more carotenoids in veraison and ‘Tinta Roriz’, ‘Trincadeira’, ‘Alicante Bouschet’, and ‘Malvasia Preta’ in maturity. In 2024 at veraison, ‘Tinta Roriz’, ‘Alicante Bouschet’, and ‘Malvasia Preta’ remained as the ones with higher content of carotenoids, while ‘Touriga Nacional’ revealed the highest value in maturity. ‘Tinto Cão’ was the variety with lower content of carotenoids in both years and in both phenological stages. Significant statistical differences were observed in the three variables under study (year (*p<* 0.05), variety (*p<* 0.001) and phenological stage (*p<* 0.001)), and in the interaction year × phenological stage × variety (*p<* 0.001).

### 3.4 Total soluble sugars and starch

The total leaf soluble sugars increased from veraison to maturity in all varieties in the growing season 2024 while in 2023 that happened only in five of the twelve varieties. The average value of the twelve varieties was quite similar in both phenological stages of 2023 and maturity 2024, and higher than in the veraison of 2024. In both years, the varieties with highest leaf sugar content (**Table 2**) were ‘Touriga Fêmea’ at veraison (45.09 mg g^-1^ in 2023 and 33.68 mg g^-1^ in 2024) and ‘Mourisco de Semente’ at maturity (40.51 mg g^-1^ in 2023 and 42.22 mg g^-1^ in 2024), while the lowest content was observed in ‘Vinhão’ (2023; 24.81 mg g^-1^) and ‘Touriga Nacional’ (2024; 16.35 mg g^-1^) at veraison, and ‘Tinto Cão’ (2023; 27.02 mg g^-1^) and ‘Alicante Bouschet’ (2024; 25.50 mg g^-1^) in maturity. Significant differences between varieties were found at both phenological stages of 2024 and in maturity of 2023 (**Table 2**).

The content of total leaf soluble sugars was influenced by the year (*p<* 0.001), phenological stage (*p<* 0.001), variety (*p<* 0.001), and by the interactions year × phenological stage (*p<* 0.001) and variety × phenological stage × year (*p<* 0.001) (**Table 2**).

The average content in leaf starch was significantly higher in both phenological stages in 2023 than in 2024 (**Table 3**). The highest average content was observed at veraison 2023, followed by maturity 2023, and then with contents much lower maturity 2024 and veraison 2024. In 2023, the starch content increased from veraison to maturity in six of the twelve varieties studied and decreased in the remaining six. In 2024, seven varieties showed an increase in leaf starch content from veraison to maturity while ‘Touriga Nacional’, ‘Trincadeira’, ‘Donzelinho Tinto’, ‘Alicante Bouschet’, and ‘Malvasia Preta’ showed a decrease. In both phenological stages of 2023, ‘Mourisco de Semente’ had the highest leaf starch content (55.06 mg g^-1^ at veraison and 34.32 mg g^-1^ at maturity), whereas ‘Tinto Cão’ had the lowest (10.26 mg g^-1^ at veraison and 12.20 mg g^-1^ at maturity). In 2024, ‘Malvasia Preta’ recorded the highest value at veraison (6.14 mg g^-1^) and ‘Touriga Fêmea’ at maturity (9.03 mg g^-1^). ‘Touriga Franca’ had the lowest content at veraison in 2024 (0.45 mg g^-1^), while ‘Touriga Nacional’ (1.28 mg g^-1^) and ‘Trincadeira’ (1.25 mg g^-1^) had the lowest values at maturity in 2024. Significant statistical differences between varieties were found in both phenological stages of the year 2023 and in maturity of 2024. Significant differences (p< 0.05) were observed between all the parameters and their interactions revealing the diversity of the twelve varieties (**Table 3**).

**Table 3:** Leaf starch content in twelve different varieties of *Vitis vinifera* in the phenological stages of veraison and maturity in the years 2023 and 2024.

Variety	Leaf starch (mg g^-1^ FW)			
	Veraison 2023	Maturity 2023	Veraison 2024	Maturity 2024
‘Touriga Nacional’	12.91 ± 1.07 a-c_1_	22.37 ± 8.04 ab_2_	2.37 ± 1.78 ns	1.28 ± 0.55 A_2_
‘Touriga Franca’	16.37 ± 0.94 c-e_1_	16.81 ± 2.27 ab_2_	0.45 ± 0.23 ns	2.09 ± 0.72 A_2_
‘Tinta Roriz’	28.25 ± 8.79 b-d_1_	19.56 ± 4.20 ab_2_	1.26 ± 0.86 ns	2.40 ± 1.66 A_2_
‘Tinto Cão’	10.26 ± 2.12 ab_1_	12.20 ± 0.35 a_2_	1.38 ± 0.39 ns	1.75 ± 1.10 A_2_
‘Tinta Barroca’	12.34 ± 1.18 ab_1_	20.22 ± 6.80 ab_2_	1.68 ± 0.98 ns	5.70 ± 1.55 AB_2_
‘Trincadeira’	11.38 ± 1.41 a-c_1_	14.34 ± 2.22 ab_2_	1.92 ± 0.66 ns	1.25 ± 0.66 AB_2_
‘Vinhão’	11.15 ± 2.09 a_1_	16.52 ± 2.21 ab_2_	1.59 ± 0.37 ns	3.30 ± 0.49 AB_2_
‘Donzelinho Tinto’	39.52 ± 4.45 de_1_	16.53 ± 3.51 ab_2_	4.42 ± 2.83 ns	1.93 ± 0.59 A_2_
‘Alicante Bouschet’	17.70 ± 3.42 a-d_1_	14.59 ± 1.81 ab_2_	4.46 ± 2.28 ns	1.79 ± 0.59 A_2_
‘Touriga Fêmea’	52.99 ± 2.89 e_1_	24.90 ± 1.92 ab_2_	4.71 ± 3.19 ns	9.03 ± 3.32 B_2_
‘Malvasia Preta’	37.53 ± 9.80 c-e_1_	13.93 ± 1.68 ab_2_	6.14 ± 3.00 ns	2.56 ± 0.62 A_2_
‘Mourisco de Semente’	55.06 ± 10.56 e_1_	34.32 ± 5.91 b_2_	1.41 ± 0.63 ns	4.15 ± 1.02 AB_2_
*p* value (V)	<0.001			
*p* value (PS)	0.033			
*p* value (Y)	<0.001			
*p* value (V × PS)	0.004			
*p* value (V × Y)	0.005			
*p* value (Y × PS)	0.027			
*p* value (V × PS × Y)	<0.001			

Values are represented as mean ± SE and the different letters and numbers represent significant differences (*p<* 0.05, Tukey’s test) between the twelve different varieties within each phenological stage (veraison – 1; maturity – 2) and each year (lowercase – 2023; uppercase – 2024).

### 3.5 Bioactive compounds

**Figure 5:**
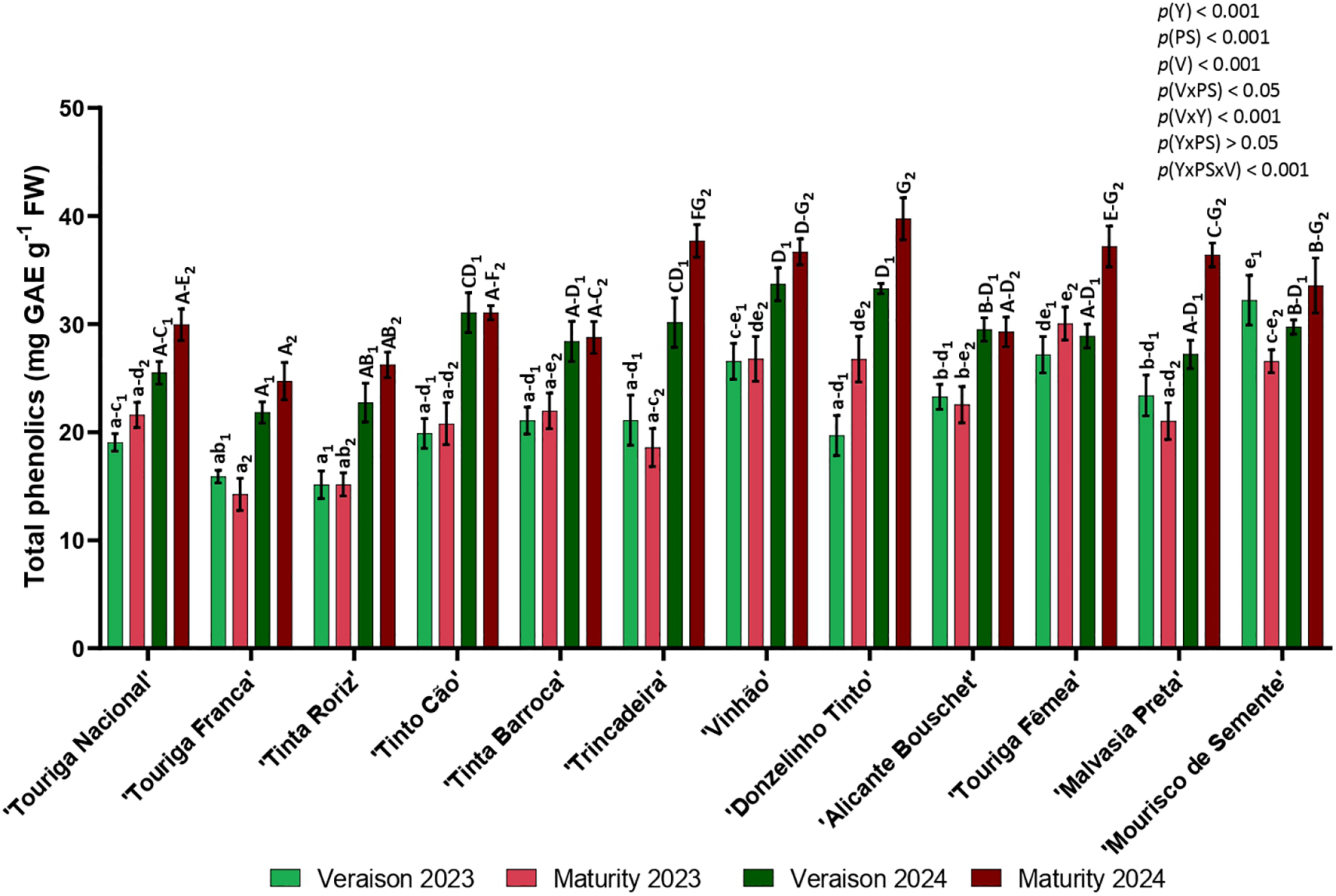
Total phenolics in twelve different varieties (V) of *Vitis vinifera* in the phenological stages (PS) of veraison and maturity in the years (Y) 2023 and 2024. Values are represented as mean ± SE and the different letters and numbers represent significant differences (*p<* 0.05, Tukey’s test) between the varieties within each phenological stage (veraison – 1; maturity – 2) and each year (lowercase – 2023; uppercase – 2024).

The bioactive compounds were determined by the total phenolics, flavonoids, and *ortho*-diphenols contents in leaf samples. **Figure 5** presents the total phenolics content which in general were higher in 2024. In most varieties, the total phenolics were higher in maturity than in veraison in both years.

Contents varied from 15.14 to 33.69 mg GAE g^-1^ in veraison (‘Tinta Roriz’, 2023 and ‘Vinhão’, 2024, respectively) and between 14.24 and 39.75 mg GAE g^-1^ in maturity (‘Touriga Franca’, 2023 and ‘Donzelinho Tinto’, 2024, respectively).

‘Mourisco de Semente’ (veraison) and ‘Touriga Fêmea’ (maturity) were the varieties with highest content in 2023 and, ‘Vinhão’ (veraison) and ‘Donzelinho Tinto’ (veraison and maturity) in 2024. On the other hand, the lowest phenolic contents were observed in ‘Tinta Roriz’ (veraison) and ‘Touriga Franca’ (maturity) in 2023, and in ‘Touriga Franca’ (both phenological stages) in 2024. From the global analysis, significant differences were obtained by the year (*p<* 0.001), variety (*p<* 0.001), and phenological stage (*p<* 0.001), for the interactions phenological stage × variety (*p<* 0.05), year × variety (*p<* 0.001), and year × phenological stage × variety (*p<* 0.001).

**Figure 6:**
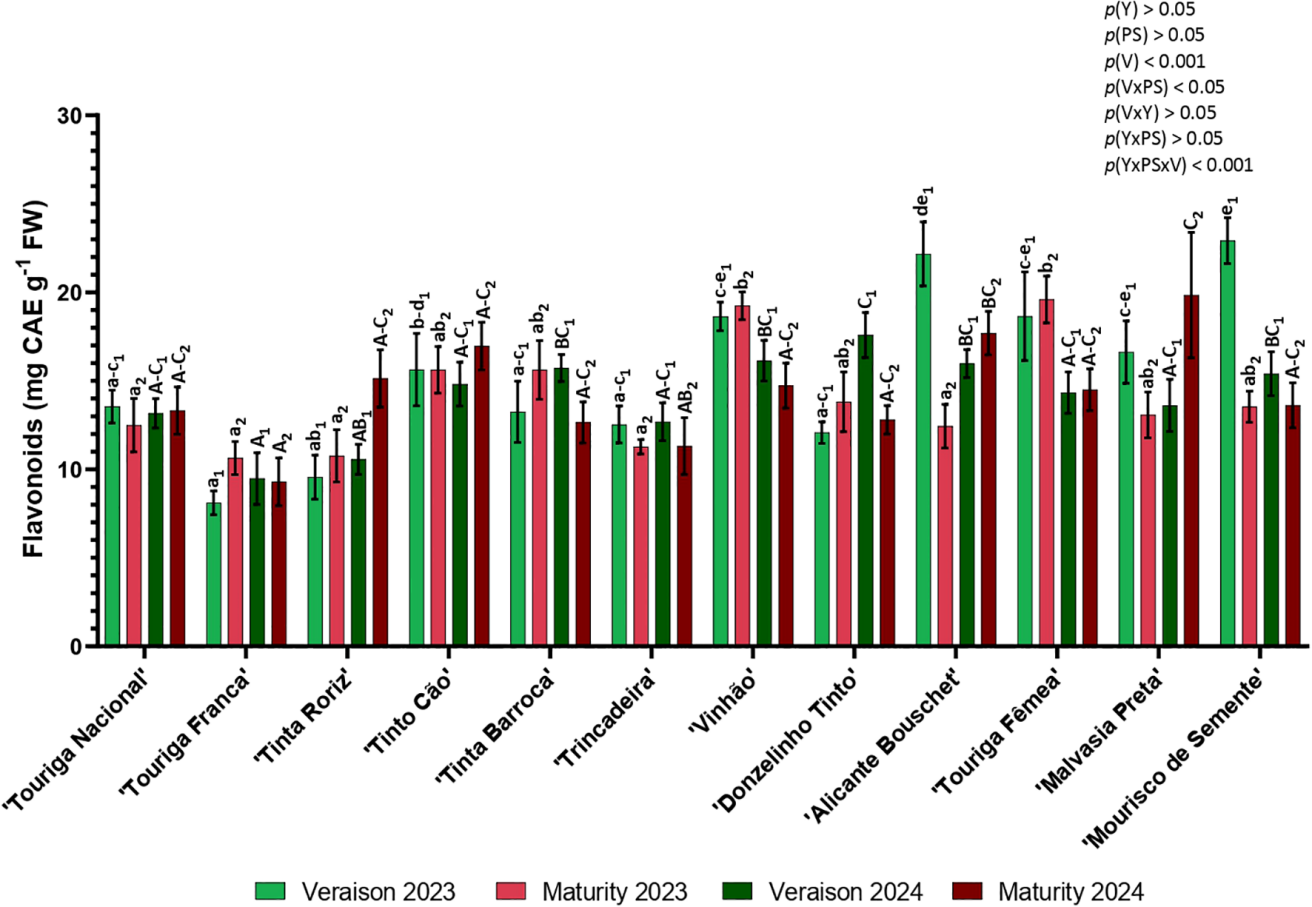
Content of Flavonoids in twelve different varieties (V) of *Vitis vinifera* in the phenological stages (PS) of veraison and maturity in the years (Y) 2023 and 2024. Values are represented as mean ± SE and the different letters and numbers represent significant differences (*p<* 0.05, Tukey’s test) between the twelve different varieties within each phenological stage (veraison – 1; maturity – 2) and each year (lowercase – 2023; uppercase – 2024).

Flavonoid concentration, presented in **Figure 6**, was higher in veraison 2023, followed by maturity 2024, veraison 2024 and lastly maturity 2023. Flavonoid content varied from 8.11 to 22.91 mg CAE g^-1^ in veraison (‘Touriga Franca’, 2023 and ‘Mourisco de Semente’, 2023, respectively) and from 9.31 to 19.85 mg CAE g^-1^ in maturity (‘Touriga Franca’, 2024 and ‘Malvasia Preta’, 2024, respectively). The flavonoid content decreased from veraison to maturity except for ‘Touriga Franca’, ‘Tinta Roriz’, ‘Tinta Barroca’, ‘Vinhão’, ‘Donzelinho Tinto’ and ‘Touriga Fêmea’ in 2023 and ‘Touriga Nacional’, ‘Tinta Roriz’, ‘Tinto Cão’, ‘Alicante Bouschet’, ‘Touriga Fêmea’ and ‘Malvasia Preta’ in 2024. The evolution of the flavonoid content was variable in 2023 and in 2024 with half of the varieties decreasing the content from veraison to maturity and half of them increasing. Eight varieties presented different behaviors in both growth seasons.

In 2023, the varieties ‘Mourisco de Semente’ and ‘Touriga Fêmea’ were the ones with highest content of flavonoids in veraison and maturity, respectively. In 2024, ‘Donzelinho Tinto’ was the one with highest content of flavonoids at veraison, while at maturity was ‘Malvasia Preta’. ‘Touriga Franca’ was the one with lowest flavonoid content in both years and both phenological stages. This phenolic compound was affected by the variety (*p<* 0.001) and the interactions phenological stage × variety (*p<* 0.05) and year × phenological stage × variety (*p<* 0.001).

**Figure 7:**
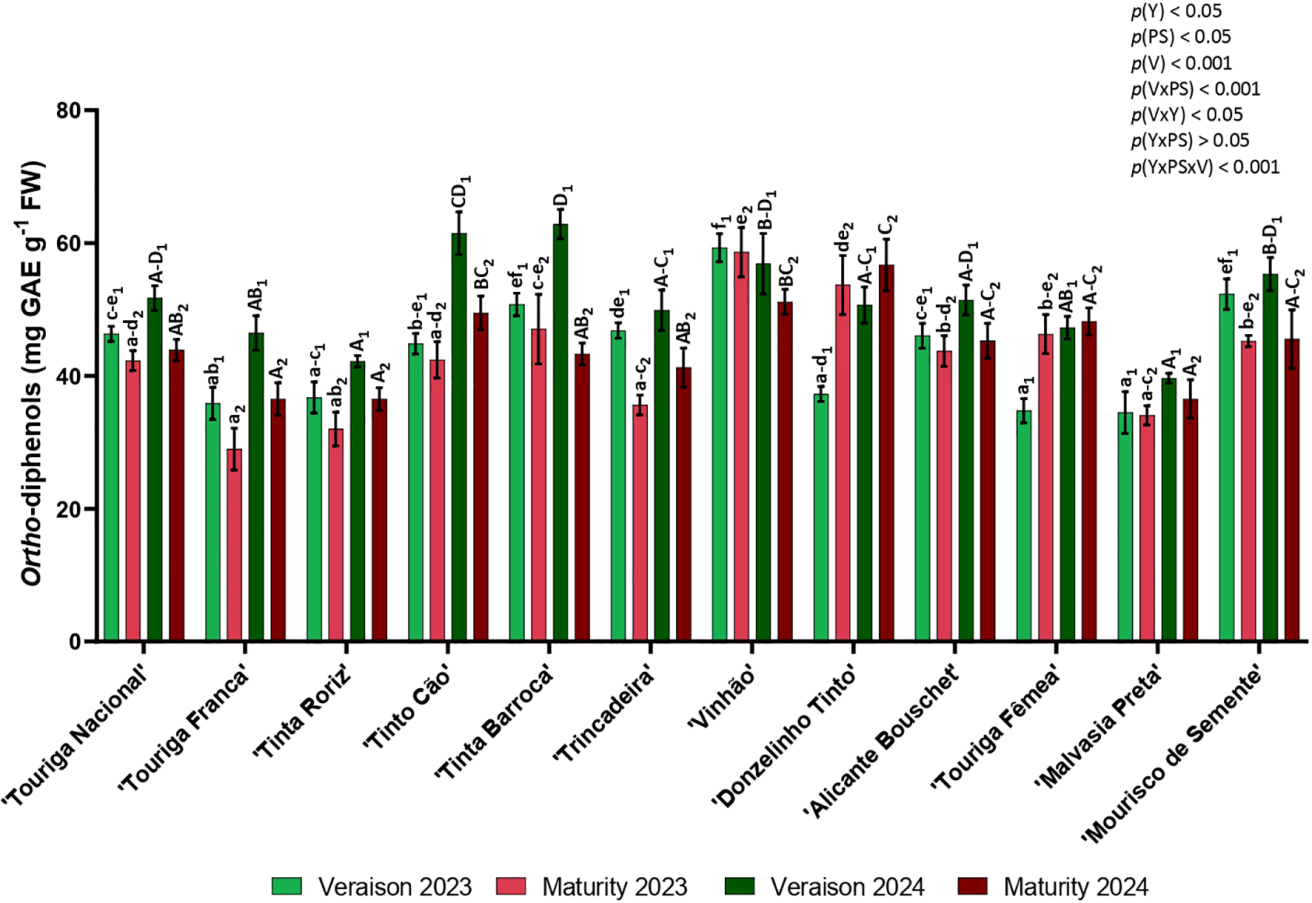
*Ortho*-diphenols in twelve different varieties (V) of *Vitis vinifera* in the phenological stages (PS) of veraison and maturity in the years (Y) 2023 and 2024. Values are represented as mean ± SE and the different letters and numbers represent significant differences (*p<* 0.05, Tukey’s test) between the twelve different varieties within each phenological stage (veraison – 1; maturity – 2) and each year (lowercase – 2023; uppercase – 2024).

In general, the *ortho*-diphenols contents were higher in 2024 than in 2023 in both phenological stages, except for ‘Tinta Barroca’ (maturity) and ‘Vinhão’ (veraison and maturity). In both years, this bioactive compound decreased from veraison to maturity in all varieties with exception of ‘Donzelinho Tinto’ and ‘Touriga Fêmea’ that registered an increase of the *ortho*-diphenol content. *Ortho*-diphenols content varied in average of the two years between 34.49 and 62.88 mg GAE g^-1^ in veraison (‘Malvasia Preta’, 2023 and ‘Tinta Barroca’, 2024, respectively) and from 28.99 to 58.64 mg GAE g^-1^ in maturity (‘Touriga Franca’, 2023 and ‘Vinhão’, 2023, respectively) (**Figure 7**). In both phenological stages of 2023, ‘Vinhão’ was the one with highest content of *ortho*-diphenols and ‘Malvasia Preta’ (veraison) and ‘Touriga Franca’(maturity) the ones with the lowest, while in 2024, ‘Tinta Barroca’ (veraison) and ‘Donzelinho Tinto’ (maturity) showed the highest value and ‘Malvasia Preta’ the lowest in both phenological stages. Significant statistical differences between varieties were found in both years and both phenological stages with emphasis in veraison. *Ortho*-diphenols were affected by the year (*p<* 0.05), the phenological stage (*p<* 0.05), the variety (*p<* 0.01), for the interactions phenological stage × variety (*p<* 0.001), year × variety (*p<* 0.05), and year × phenological stage × variety (*p<* 0.001).

### 3.6 Antioxidant activity

Regarding the antioxidant activity three different assays were determined, ABTS, DPPH and FRAP. In general, the ABTS was higher in 2023 than in 2024, namely in five varieties at veraison and another five at maturity. Only the varieties ‘Touriga Fêmea’ (veraison) and ‘Mourisco de Semente’ (maturity) registered slightly higher values. In the year 2023, the content of ABTS varied between 12.42 and 38.60 mg Trolox g^-1^ in veraison (‘Tinto Cão’ and ‘Tinta Roriz’, respectively) and from 10.37 and 43.92 mg Trolox g^-1^ in maturity (‘Touriga Fêmea’ and ‘Touriga Franca’, respectively). In 2024, ‘Tinto Cão’ had the lowest ABTS content in veraison with 12.42 mg Trolox g^-1^ and ‘Touriga Franca’ had the highest with 30.10 mg Trolox g^-1^. In maturity, ‘Vinhão’ had the lowest ABTS content and ‘Touriga Franca’ the highest with 16.68 and 41.68 mg Trolox g^-1^, respectively.

**Figure 8:**
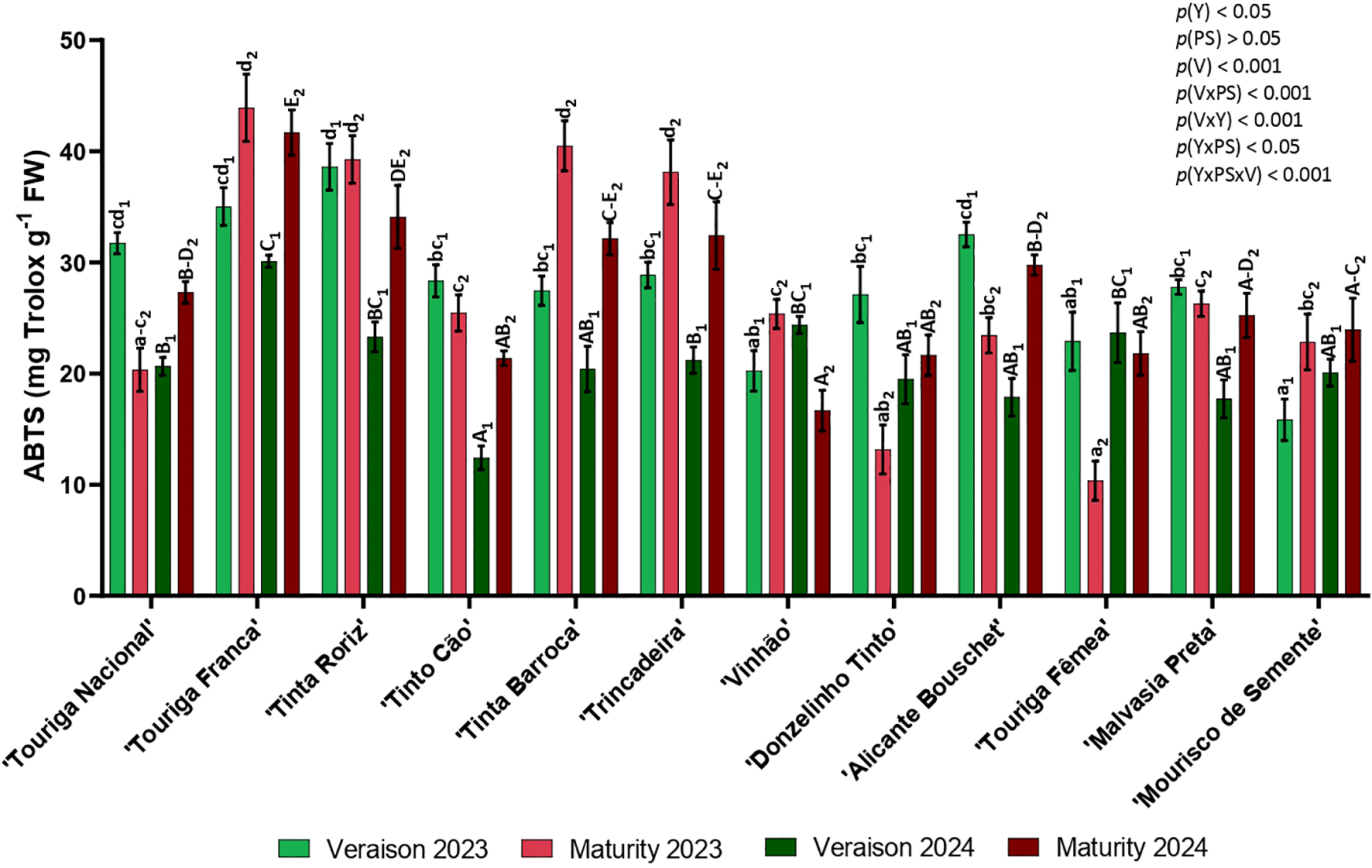
Antioxidant activity: ABTS radical-scavenging activity in twelve different varieties (V) of *Vitis vinifera* in the phenological stages (PS) of veraison and maturity in the years (Y) 2023 and 2024. Values are represented as mean ± SE and the different letters and numbers represent significant differences (*p<* 0.05, Tukey’s test) between the twelve different varieties within each phenological stage (veraison – 1; maturity – 2) and each year (lowercase – 2023; uppercase – 2024).

In ABTS data, it was observed a significant influence of the year (*p<* 0.05), the variety (*p<* 0.001), the interactions phenological stage × variety (*p<* 0.001), year × variety (*p<* 0.001), year × phenological stage (*p<* 0.05), and year × phenological stage × variety (*p<* 0.001) (**Figure 8**).

**Figure 9:**
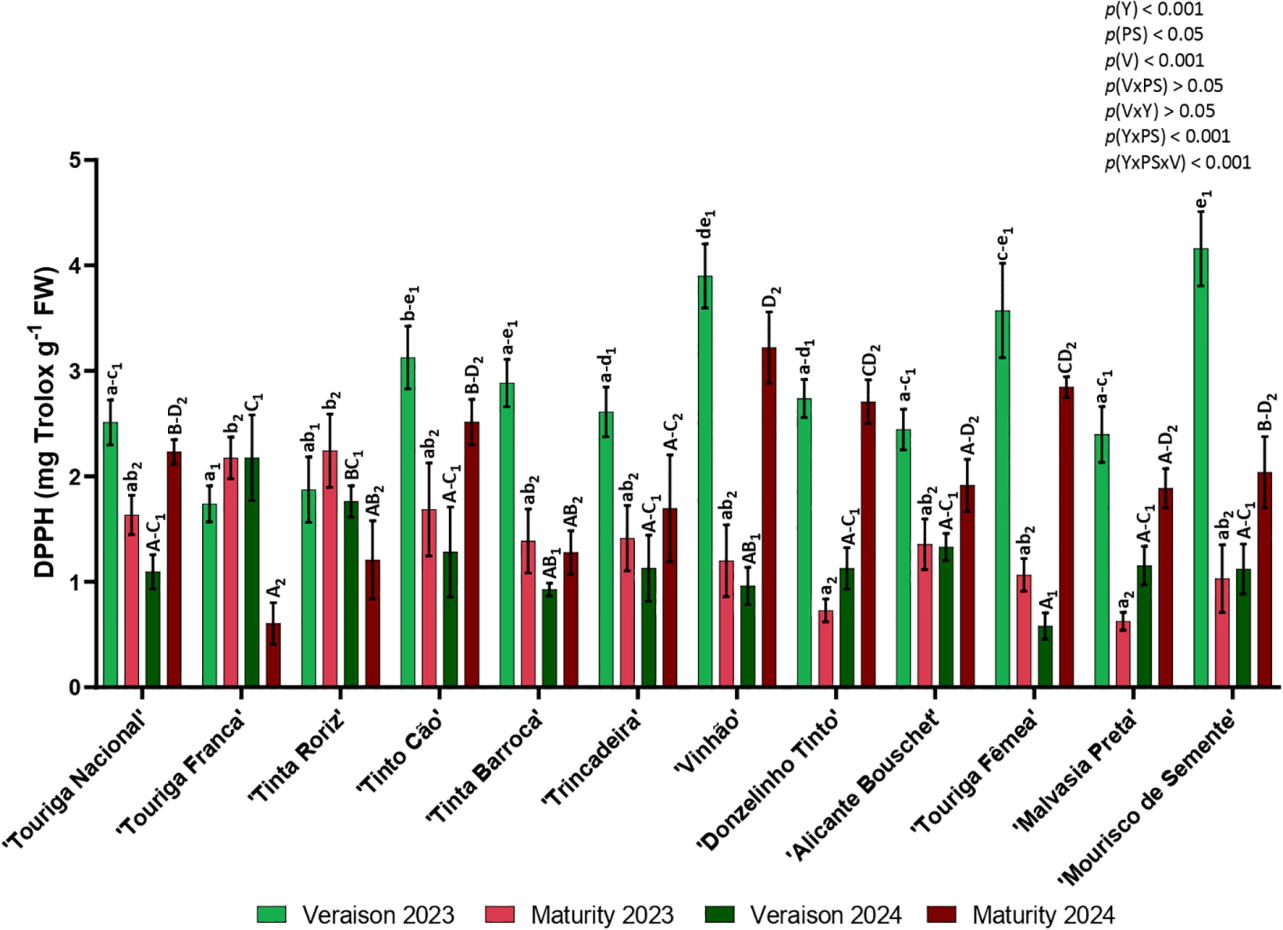
Antioxidant activity: DPPH radical-scavenging activity in twelve different varieties (V) of *Vitis vinifera* in the phenological stages (PS) of veraison and maturity in the years (Y) 2023 and 2024. Values are represented as mean ± SE and the different letters and numbers represent significant differences (*p<* 0.05, Tukey’s test) between the twelve different varieties within each phenological stage (veraison – 1; maturity – 2) and each year (lowercase – 2023; uppercase – 2024).

The DPPH activity (**Figure 9**) was higher in veraison 2023, following maturity 2024 in nine of the twelve varieties. In 2023, the varieties with highest DPPH activity were ‘Mourisco de Semente’ (4.19 mg Trolox g^-1^) in veraison and ‘Tinta Roriz’ (2.24 mg Trolox g^-1^) in maturity. The varieties with lower activity were ‘Touriga Franca’ (1.74 mg Trolox g^-1^) in veraison and ‘Malvasia Preta’ (0.63 mg Trolox g^-1^) in maturity. In 2024, ‘Touriga Franca’ (2.18 mg Trolox g^-1^) had the highest DPPH activity in veraison and ‘Vinhão’ (3.22 mg Trolox g^-1^) in maturity. The varieties with the lowest activity from the same year were ‘Touriga Fêmea’ (veraison, 0.58 mg Trolox g^-1^) and ‘Touriga Franca’ (maturity, 0.61 mg Trolox g^-1^). DPPH radical-scavenging activity assay showed significant differences in the year (*p<* 0.001), the phenological stage (*p<* 0.05), the variety (*p<* 0.001), and the interactions year × phenological stage (*p<* 0.001) and year × phenological stage × variety (*p<* 0.001).

**Figure 10:**
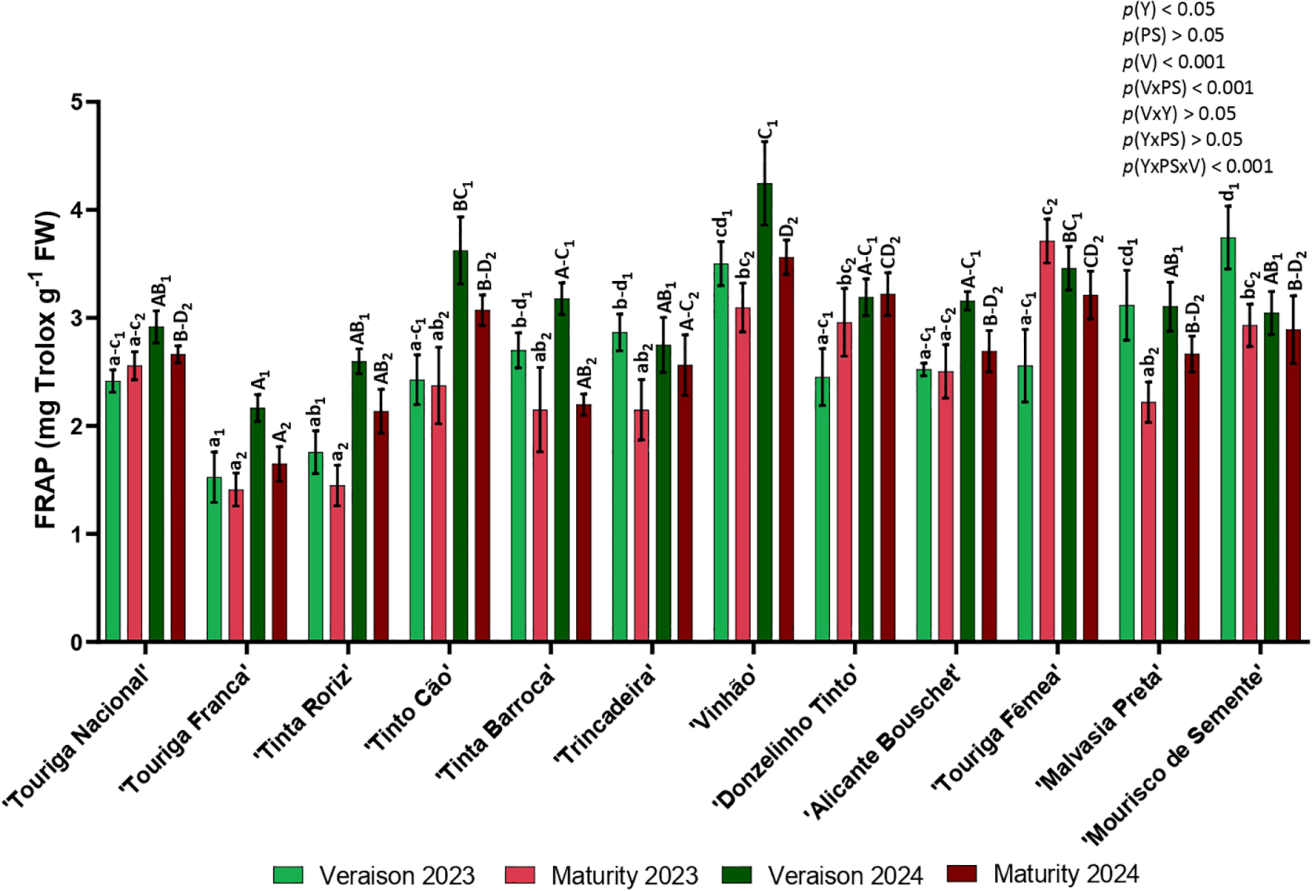
Antioxidant activity: FRAP assay in twelve different varieties (V) of *Vitis vinifera* in the phenological stages (PS) of veraison and maturity in the years (Y) 2023 and 2024. Values are represented as mean ± SE and the different letters and numbers represent significant differences (*p<* 0.05, Tukey’s test) between the twelve different varieties within each phenological stage (veraison – 1; maturity – 2) and each year (lowercase – 2023; uppercase – 2024).

In general, FRAP assay presented higher values in 2024 than in 2023, being veraison the phenological stage that showed higher levels in this antioxidant assay (**Figure 10**). In 2023, ‘Mourisco de Semente’ had the highest FRAP activity in veraison (3.75 mg Trolox g^-1^) and ‘Touriga Fêmea’ in maturity (3.71 mg Trolox g^-1^). ‘Vinhão’ had the highest FRAP activity in both phenological stages of 2024 (4.25 mg Trolox g^-1^ at veraison and 3.56 mg Trolox g^-1^ at maturity). ‘Touriga Franca’ was the variety with lowest FRAP in both years at veraison (1.53 mg Trolox g^-1–^2023 and 2.17 mg Trolox g^-1^ 2024) and maturity (1.41 mg Trolox g^-1–^2023 and 1.65 mg Trolox g^-1^ 2024). The data obtained revealed significant differences on the year (*p<* 0.05), the variety (*p<* 0.001), and the interactions phenological stage × variety (*p<* 0.001) and year × phenological stage × variety (*p<* 0.001).

### 3.7 Integrative analysis to select the most promising grapevine varieties

**Figure 11:**
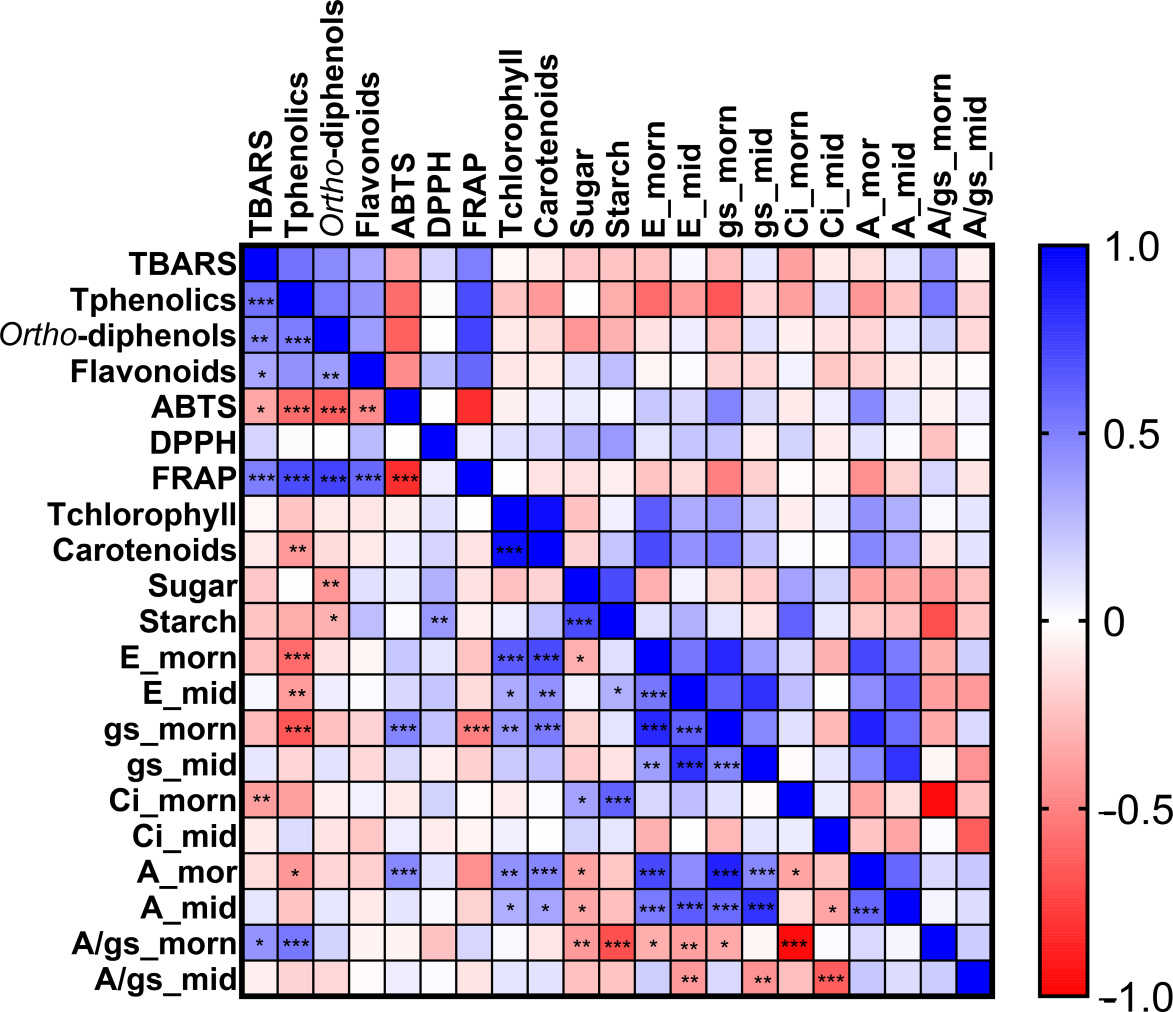
Pearson correlation among the biochemical and physiological parameters in the twelve grapevine varieties evaluated in this study. Positive correlations were indicated in blue and negative correlations were indicated in red; the *, **, and *** correspond to *p<* 0.05, *p<* 0.01, and *p<* 0.001, respectively.

A Pearson correlation was performed revealing significant positive and negative correlation (*p* < 0.05) among several of the investigated parameters (**Figure 11**).

Highly significant positive correlations were detected between several leaf gas exchange measurements at morning and biochemical parameters such as E, g_s_, and A with the content of carotenoids, Ci with leaf starch, A and g_s_ with antioxidant activity ABTS, and A/gs with phenolics. Likewise, the antioxidant activity FRAP was positive correlated with total phenolics, *ortho*-diphenols, and flavonoids. Conversely, significant negative correlations were observed between the gas exchange parameters in the morning E and g_s_ with total phenolics and gs and A/g_s_ with FRAP.

**Figure 12:**
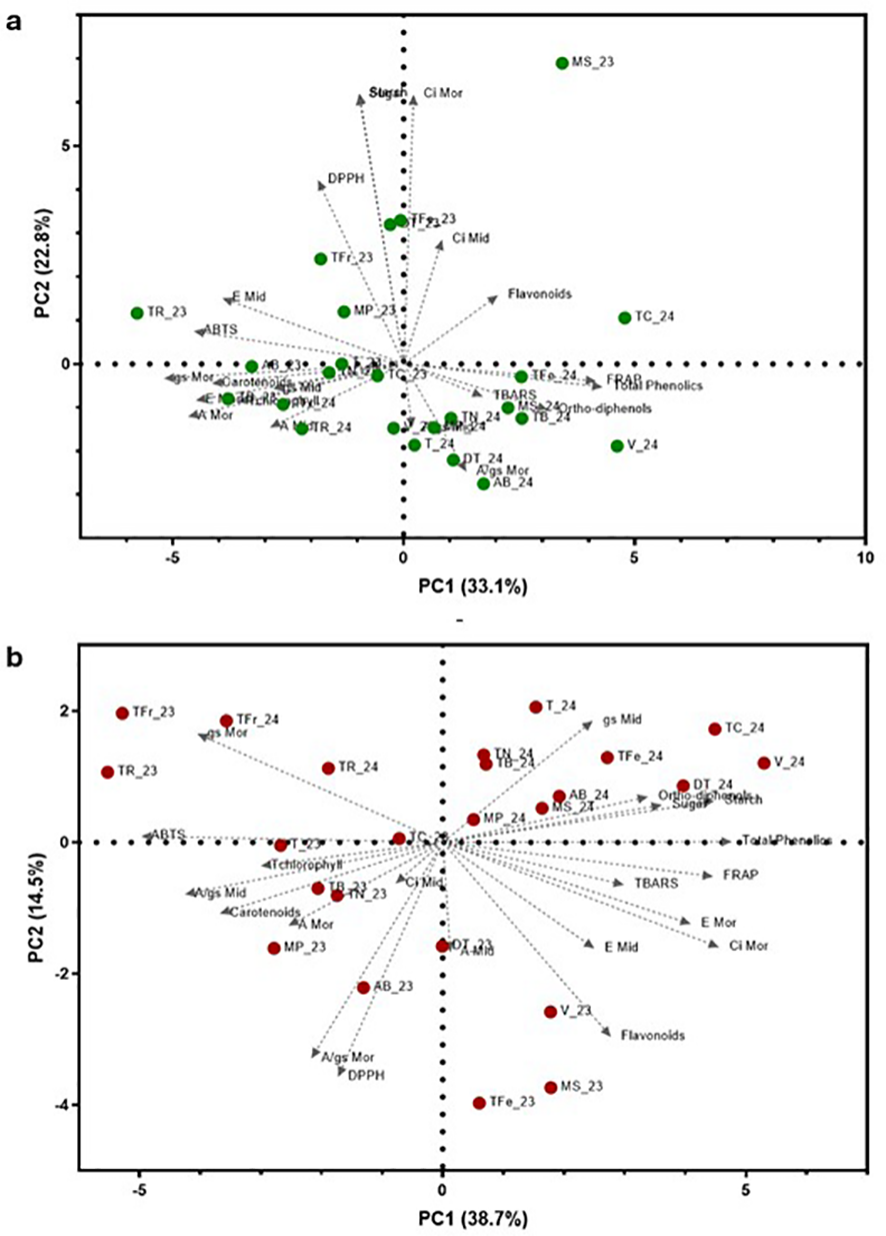
Principal component analysis based on all the physiological and biochemical parameters evaluated in the twelve grapevine varieties in two consecutive years, 2023 and 2024, and in two phenological stages, veraison and maturarion. **(a)** PCA with varieties at veraison stage and the contributions of each parameter in both years; **b)** PCA with varieties at maturation stage and the contributions of each parameter in both years. TN, ‘Touriga Nacional’; TFr, ‘Touriga Franca’; TR, ‘Tinta Roriz’; TC, ‘Tinto Cão’; TB, ‘Tinta Barroca’; T, ‘Trincadeira’; V, ‘Vinhão’; DT, ‘Donzelinho Tinto’; AB, ‘Alicante Bouschet’; TFe, ‘Touriga Fêmea’; MP, ‘Malvasia Preta’; MS, ‘Mourisco de Semente’.

Principal component analysis (PCA) was carried out on the twelve grapevine varieties (in two consecutive years and two phenological stages) and twenty-one physiological and biochemical traits with the objective of inferring the grapevine varietal distribution and behavior under summer stress conditions (**Figure 12**). For the veraison stage, the first two components of PCA accounted for 55.9% (PC1 = 33.1% and PC2 = 22.8%) of the loading scores for the all the parameters evaluated, while for the maturation stage was obtained 53.2% (PC1 = 38.7% and PC2 = 14.5%) (**Table 4**).

**Table 4:** Contribution of the first two principal component axis to variation in the twenty-one parameters used for the evaluation of twelve grapevine varieties in two consecutive years, 2023 and 2024, and two phenological stages, veraison, and maturation.

Variables	PC1	PC2
TBARS	0.39615	-0.50309
TPhenolics	**0.79831**	-0.34512
*Ortho*-diphenols	0.41233	-0.48009
Flavonoids	0.34434	-0.05081
ABTS	-0,55284	0.19834
DPPH	-0.12986	0.18759
FRAP	0.63149	-0.28204
Tchlorophyl	-0.53062	-0.28658
Carotenoids	-0.65225	-0.17830
Sugar	0.11508	**0.76491**
Starch	-0.18611	**0.76521**
E_morn	-0.83960	-0.18962
E_mid	-0.66207	-0.02707
gs_morn	-**0.92071**	-0.10380
gs_mid	-0.52913	-0.31625
Ci_morn	-0.16902	0.73063
Ci_mid	0.15648	0.28437
A_mor	-0.77128	-0.45514
A_mid	-0.60155	-0.52085
A/gs_morn	0.36135	**-0.72956**
A/gs_mid	-0.09758	-0.27260
Eigenvalues	**5.98**	**3.89**
% Variance	**28.50**	**18.50**

Values highlighted in bold represent the highest contributions.

In both PCAs (**Figures 12a, b**), no clear distribution of grapevine varieties was observed; however, a slight clustering of the samples based on the year of the experiment was detected. A total of two independent groups were obtained for this analysis (A and B, **Figure 12b**). At veraison, total phenolics (0.83) and morning g_s_ (-0.91) exhibited the highest loadings on PC1, while starch content (0.87) and morning A/g_s_ (-0.86) were the main contributors to PC2. For other hand, at maturation, total phenolics (0.91) and ABTS (-0.81) presented the highest loadings on PC1, while midday g_s_ (0.55) and DPPH (-0.86) were the main contributors to PC2.

## 4 Discussion

The exposure of plants to summer stress under field conditions affects their responses at multiple levels, triggering a range of mechanisms that enhance their ability to tolerate stress (Chaves et al., 2010). Grapevines can present different behaviors in their response to stress, displaying either isohydric or anisohydric strategies. This response may even change within the same variety when exposed to different environmental conditions (Hochberg et al., 2013). Isohydric varieties close their stomata to reduce transpiration and maintain a more constant leaf water potential becoming more tolerant to drought in mild water stress, whereas anisohydric varieties tend to keep their stomata open to maintain photosynthetic rates, being more tolerant to drought under severe water stress (Baltazar et al., 2025a). In this study, leaf gas exchange measurements were analyzed to gain insights into the adaptation and stability of different varieties under summer stress conditions (Monteiro et al., 2024b). In 2023 ‘Touriga Franca’, and in 2024 ‘Tinto Cão’, ‘Vinhão’, and ‘Touriga Fêmea’, presented a more anisohydric behavior. These results highlight that varying environmental conditions can lead to different behavioral patterns in the same varieties from year to year (Baltazar et al., 2025a) being fundamental a further investigation in future growing seasons. In 2023 there was higher precipitation and a larger temperature range than in 2024. The year 2024 had the highest maximum temperature of both years, whereas 2023 had the lowest minimum temperature.

Some studies refer that exposure to summer stress, particularly high temperatures, can lead to changes in carbohydrate metabolism in grapevines, including the accumulation of total soluble sugars and starch in leaves (Bernardo et al., 2018; Baltazar et al., 2025a). Under stress conditions, such as water or summer stress, reduced sugar and impaired starch degradation may result in starch overaccumulation in chloroplasts (Patakas and Noitsakis, 2001; Monteiro et al., 2024b). In the present study, the varieties ‘Touriga Franca’ and ‘Donzelinho Tinto’ showed an increase in leaf sugar and starch contents from veraison to maturity in 2024, which is consistent with stress-induced limitations in carbohydrate translocation rather than enhanced photosynthetic activity. This achievement is supported by the negative correlation observed between photosynthesis and leaf sugar content (**Figure 11**).

Summer stress can influence the content of photosynthetic pigments (Monteiro et al., 2024b) and the variation in photosynthetic pigment contents among varieties may also represent an adaptation strategy to cope with stress. According to Moutinho Pereira et al (Moutinho-Pereira et al., 2007), the lower chlorophyll content observed in ‘Tinto Cão’, in comparison to other varieties, may represent an adaptation strategy aimed at mitigating the impacts of higher radiation levels and elevated temperatures (Moutinho-Pereira et al., 2007). Chlorophyll content generally decreased over the season, especially under summer stress conditions, as shown in **Figure 3**. An average reduction of 30.4% in chlorophyll content was observed between veraison and maturity. The variety ‘Tinta Barroca’ (62.6%) in 2023 and ‘Tinta Roriz’ (56.6%) in 2024 showed the greatest losses, while ‘Alicante Bouchet’ (5.6%) in 2023 and ‘Touriga Nacional’ (4.4%) in 2024 presented the lowest reductions. Carotenoids play a key role in protecting plants from photooxidative damage by reducing the formation of reactive oxygen species (ROS), which can lead to lipid peroxidation, irreversible DNA damage, and even cell death (Baltazar et al., 2025a). In this study, several varieties presented high carotenoid contents across both years and phenological stages, such as ‘Tinta Barroca’, ‘Vinhão’, ‘Donzelinho Tinto’, ‘Alicante Bouschet’ and ‘Malvasia Preta’. However, ‘Tinto Cão’ consistently showed the lowest content of carotenoids in both years and at both phenological stages, in agreement with the findings of Moutinho Pereira et al (Moutinho-Pereira et al., 2007).

The increase of lipid peroxidation levels in leaves can be a response to summer stress in grapevine varieties (Monteiro et al., 2024a). In both years and phenological stages, ‘Touriga Franca’ consistently presented the lowest TBARS content, indicating the lowest level of lipidic peroxidation. Bernardo et al. (2021) used TBARS quantification to explore the level of stress in ‘Touriga Nacional’ and ‘Touriga Franca’ and this one also presented lower content. On the other hand, ‘Vinhão’, ‘Donzelinho Tinto’, ‘Alicante Bouschet’ presented the higher levels of TBARS which may suggest more susceptibility to summer stress.

Secondary metabolites such as phenolic compounds play an important role in berries and wine quality traits. Due to their antioxidant activity, they also contribute to neutralizing ROS (Monteiro et al., 2024a). The exploitation of phenolic compounds in grapevine leaves has interest due to the information about vine vigor, senescence, stress due to water scarcity or occurrence of fungal diseases (Anić et al., 2024). In grapevine leaves, the synthesis of phenolic compounds can be triggered by stress conditions, such as drought and increased UV radiation. However, these contents tend to decrease in later stages as the leaves enter senescence (Anić et al., 2024).

In general, the total phenolic content of the studied varieties increased in maturity, which can be explained by the increase in stress that the plant is subjected to. This induces the expression of phenylalanine ammonia-lyase (PAL) involved in the phenylpropanoid pathway that leads to an increase of total phenolics (Wen et al., 2005; Zhao et al., 2021).

Regarding flavonoids, the results were only influenced by variety. ‘Touriga Franca’ had the lowest flavonoid content across both years and phenological stages, as expected, since flavonoids are known to be involved in the protection against stress (Monteiro et al., 2024b) and ‘Touriga Franca’ presented the lowest TBARS content of all the varieties. Conversely, varieties previously identified as more susceptible to stress, such as ‘Touriga Fêmea’, exhibited higher flavonoid levels that can be interpreted as a stress response aimed at mitigating oxidative damage (Anić et al., 2024). For *ortho*-diphenols, higher levels are typically observed at veraison and lower levels at maturity, since the plant synthesizes these compounds during veraison to cope with stress. During maturity, however, the plant tends to prioritize resource allocation to the berries rather than the leaves, which may explain the observed decrease in *ortho*-diphenols at this stage (Król et al., 2014; Anić et al., 2024). The results of the antioxidant activity assays (ABTS, DPPH, and FRAP) varied throughout the vegetative cycle of the grapevine and differed between assays, which is expected given that each assay targets different molecules (Shah and Modi, 2015). It is important to refer that long-term experiments are important to consolidate the data obtained for phenolic compounds and antioxidant activity.

Overall, the antioxidant activity assessed by ABTS, DPPH, and FRAP assays was closely associated with the content and profile of phenolic compounds in grapevine leaves. Varieties and phenological stages exhibiting higher total phenolics, flavonoids, and particularly ortho-diphenols generally showed enhanced antioxidant capacity, reinforcing the well-established role of phenolic compounds as major contributors to antioxidant activity. *Ortho*-diphenols, due to their chemical structure with adjacent hydroxyl groups, are especially effective radical scavengers and metal chelators, which may explain their strong relationship with ABTS and FRAP responses.

The higher antioxidant activity observed mainly in veraison 2023 and maturity 2024 for several varieties is consistent with the elevated levels of specific phenolic subclasses at these stages, suggesting a dynamic regulation of secondary metabolism in response to phenological development and interannual climatic variability. Differences among varieties further highlight the strong genetic influence on both phenolic composition and antioxidant capacity. Although some discrepancies among assays were observed, these are expected due to the different reaction mechanisms involved, with ABTS and DPPH reflecting radical-scavenging capacity and FRAP measuring reducing power. Collectively, the results indicate that the antioxidant activity of grapevine leaves is largely driven by their phenolic composition, particularly by ortho-diphenols and flavonoids, whose relative contribution varies with variety, phenological stage, and year.

The study and analysis of grapevine varietal diverse physiological and biochemical responses is a challenging task, as has been referred by Khandani et al. (2024) (Khandani et al., 2024). This study revealed highly significant correlations between gas exchange and biochemical parameters such as carotenoids with E, g_s_ and A, the last two with the antioxidant activity ABTS and also A/g_s_ with phenolics.

Integrating all the results from the study, it is possible to suggest which varieties are better suited to withstand summer stress. ‘Tinta Roriz’ presented the most stable physiological response to summer stress in both years, maintaining high photosynthetic activity early in the season and moderate antioxidant capacity through maturity. This ability to preserve both carbon assimilation and oxidative balance has been associated with increased resilience in grapevine varieties under stress (Hochberg et al., 2013; Hochberg et al., 2017). ‘Alicante Bouschet’ in both years presented exceptional early antioxidant protection. Even though it showed some physiological decline by maturity it maintained enough phenolic activity to counteract oxidative stress, being considered as a variety with higher tolerance to summer stress than most other varieties in this study. Varieties with strong antioxidant systems are better equipped to detoxify ROS and maintain cell membrane integrity (Agati et al., 2012). Even though this variety presented a high TBARS content, stress tolerance is not defined by the presence of stress markers but by the plants’ ability to respond and recover from this stress with effective antioxidant defenses (Chaves et al., 2009; Suzuki et al., 2012). ‘Tinta Barroca’ and ‘Tinto Cão’ presented a moderate to lower summer stress tolerance with a modest performance at the beginning of the season but failed to improve or compensate when the exposure to summer stress increased. ‘Tinta Barroca’ showed a better tolerance in 2023 with its defenses and function more weakened in 2024. ‘Tinto Cão’ remained weak in both functional and antioxidant traits in both years of the study. ‘Touriga Nacional’ and ‘Trincadeira’ can be considered moderately tolerant to summer stress in both years with good leaf gas exchange and photosynthetic pigments in early season but declined by maturity. The antioxidant parameters were median at veraison but also decreased at maturity. ‘Donzelinho Tinto’ showed a lower stress tolerance in 2023 than in 2024. In 2024, it showed intermediate physiological performance at veraison, together with relatively high antioxidant capacity that declined toward maturity, but remained higher than in the most stress susceptible varieties, supporting its classification as having moderate tolerance to summer stress. ‘Touriga Franca’ showed highest tolerance to stress in 2024 than in 2023. It maintained a high photosynthetic activity in both years but a low biochemical defense, being considered moderately tolerant to summer stress. Seeing that ‘Touriga Franca’ presented the lowest TBARS levels, but a low antioxidant defense, it might suggest that this variety relies on other non-phenolic or enzymatic antioxidant systems (Suzuki et al., 2012) to buffer ROS without the need for high phenolic investment. ‘Touriga Fêmea’, ‘Malvasia Preta’, and ‘Mourisco de Semente’ presented as the most sensitive varieties to summer stress with low photosynthetic rates, limited antioxidant activity, and reduced photosynthetic pigments. The low water use efficiency, and the decrease in gas exchanges are usually associated with lower productivity under stress (Medrano et al., 2002).

## 5 Conclusion

The results of this study clearly demonstrate that grapevine varieties respond differently to summer stress, with the behavior of each variety being influenced by the specific environmental conditions experienced each year. The variables studied indicate that the response to stress is not uniform, and the adaptability of each variety can be strongly conditioned by climatic factors such as high temperatures and intense solar radiation. Among the varieties most sensitive to summer stress, ‘Malvasia Preta’, ‘Touriga Fêmea’, and ‘Mourisco de Semente’ stood out, exhibiting higher levels of lipid peroxidation, lower carotenoid and flavonoid content, and reduced antioxidant capacity. These results suggest that these varieties may face greater challenges under stress conditions, potentially affecting their yield and quality in the future context of viticulture.

On the other hand, the varieties ‘Tinta Roriz’, ‘Alicante Bouschet’, and ‘Vinhão’ demonstrated a more robust physiological profile in response to stress, with a superior ability to accumulate antioxidant compounds such as phenolics and flavonoids, as well as maintain membrane integrity. This suggests a higher tolerance to summer stress. These varieties not only displayed greater stress resilience but also indicated greater potential for long-term viticulture, particularly in the context of climate change.

This study highlights the importance of selecting grapevine varieties that are better adapted to the increasing stress conditions driven by climate change, such as a strategy to help mitigate the negative impacts of global warming and extreme environmental events. The use of stress-resilient varieties could not only enhance grape yield and quality but also contribute to the long-term sustainability of viticulture in regions vulnerable to high temperatures and other adverse climatic factors.

## Data Availability

The original contributions presented in the study are included in the article/supplementary material. Further inquiries can be directed to the corresponding author.
